# MIS416 Enhances Therapeutic Functions of Human Umbilical Cord Blood-Derived Mesenchymal Stem Cells Against Experimental Colitis by Modulating Systemic Immune Milieu

**DOI:** 10.3389/fimmu.2018.01078

**Published:** 2018-05-28

**Authors:** Byung-Chul Lee, Nari Shin, Jin Young Lee, Insung Kang, Jae-Jun Kim, Seung Eun Lee, Soon Won Choi, Gill A. Webster, Kyung-Sun Kang

**Affiliations:** ^1^Adult Stem Cell Research Center, College of Veterinary Medicine, Seoul National University, Seoul, South Korea; ^2^College of Veterinary Medicine, Research Institute for Veterinary Science, Seoul National University, Seoul, South Korea; ^3^Innate Therapeutics Ltd., AMSC, Auckland, New Zealand

**Keywords:** stem cell therapy, NOD2 and TLR9 signaling, immune modulator, inflammatory bowel diseases, migration capacity, immunosuppression

## Abstract

Human adult stem cells, including umbilical cord blood-derived mesenchymal stem cells (hUCB-MSCs), have recently been considered a promising alternative treatment for inflammatory bowel disease (IBD) due to their unique immunomodulatory properties and ability to promote tissue regeneration. However, despite many years of research and pre-clinical studies, results from clinical trials using these cells have been diverse and conflicting. This discrepancy is caused by several factors, such as poor engraftment, low survival rate, and donor-dependent variation of the cells. Enhancement of consistency and efficacy of MSCs remains a challenge for the feasibility of cell-based therapy. In this study, we investigated whether administration of MIS416, a novel microparticle that activates NOD2 and TLR9 signaling, could enhance the therapeutic efficacy of hUCB-MSCs against Crohn’s disease, using dextran sulfate sodium (DSS)-induced colitis model. Colitis was experimentally induced in mice by using 3% DSS, and mice were administered a retro-orbital injection of MIS416 and subsequent intraperitoneal injection of hUCB-MSCs. Mice were examined grossly, and blood, spleen, and colon tissues were subsequently collected for further *ex vivo* analyses. To explore the effects of MIS416 on the therapeutic process, hUCB-MSCs and primary isolated immune cells were cultured with MIS416, and *in vitro* assays were performed. Compared to the single administration of hUCB-MSCs, co-administration with MIS416 improved the therapeutic efficiency of the stem cells by significantly alleviating the symptoms of IBD. Interestingly, MIS416 did not exert any direct effect on the immunomodulatory capacity of hUCB-MSCs. Instead, systemically injected MIS416 altered the immune milieu in the colon which caused hUCB-MSCs to be more readily recruited toward the lesion site and to suppress inflammation more efficiently. In addition, considerable numbers of regulatory immune cells were stimulated as a result of the cooperation of MIS416 and hUCB-MSCs. These findings indicate that co-administration with MIS416 enhances the therapeutic potential of hUCB-MSCs by systemically regulating the immune response, which might be an effective strategy for overcoming the current obstacles to stem cell therapy in clinical practice.

## Introduction

Inflammatory bowel diseases (IBDs), including Crohn’s disease and ulcerative colitis, are chronic relapsing disorders characterized by excessive intestinal inflammation. Although the precise etiology of IBDs remains unclear, it is known that several causes are involved in the disease onset. The concerted action of genetic susceptibility, environmental risk factors, and alterations of the microbiota triggers dysregulated immune responses, resulting in the impairment of mucosal barrier functions. Conventional treatments for IBDs, including antibiotics, anti-inflammatory drugs, and immunosuppressive medicines have limitations such as drug resistance and low therapeutic responses in certain groups of patients. To overcome these limitations, alternative therapies, such as probiotics, anti-tumor necrosis factor (TNF) therapy, and transplantation of MSCs have recently emerged (1, 2). Among these remedies, MSCs have been studied for IBD treatment because of their immunomodulatory properties, tissue regenerative capacity, and ability to migrate toward damaged areas. Key immunomodulatory function of MSCs that have been demonstrated both *in vitro* and *in vivo* is their ability to inhibit the excessive proliferation and maturation of immune cells (3).

Although the therapeutic use of human adult stem cells, including umbilical cord blood-derived mesenchymal stem cells (hUCB-MSCs) has been investigated for decades, standardization issues remain to be overcome. For example, reduced productivity of MSCs caused by replicative senescence and donor-to-donor variations make it difficult to maintain consistent therapeutic effects for each recipient (4). Several strategies have recently been investigated for enhancement of the therapeutic potential of MSCs. Previously, we reported that NOD2 activation through muramyl dipeptide (MDP) priming upregulated prostaglandin E_2_ (PGE_2_) secretion from hUCB-MSCs and increased anti-inflammatory effects in experimental models of IBD (5). Similarly, priming of MSCs with growth factors or cytokines has also been reported (6). However, these methods have not been fully verified with regards to safety or optimization. Although many investigations have been performed to elaborate these strategies, other simplified methods are still needed for convenient application.

MIS416 is a novel immunomodulatory microparticle derived from *Propionibacterium acnes*, which consists of MDP and bacterial DNA. Phagocytic cells, key responders to MIS416, internalize MIS416, resulting in the activation of cytoplasmic receptors, NOD2 and TLR9 (7). NOD2 and TLR9-dependent pathways have been highlighted as therapeutic targets in IBDs, as NOD2 and TLR9 dysfunctions have been shown to play a central role in disease pathophysiology. For example, a frame-shift mutation of NOD2 was associated with the development of Crohn’s disease in a study of over 400 unrelated subjects (8), and in experimental colitis, a NOD2 deficiency exacerbated disease severity due to an uncontrolled immune response leading to immune hyper-responsiveness to intestinal antigens mediated by IL-12-producing antigen-presenting cells (9). In contrast, overexpression of the NOD2 gene rescued mice from peptidoglycan-induced colitis (10), and mice deficient in TLR9 and MyD88 no longer demonstrated probiotic-mediated inhibitory effects on intestinal inflammation in experimental colitis (11). Furthermore, TLR9-induced type I interferon (IFN) resolved intestinal inflammation, and this effect was abolished by type I IFN neutralizing antibodies (12). As a NOD2 and TLR9 agonist, MIS416 has the potential to immune modulate IBDs by distinct and complimentary mechanisms. As well as inducing expansion of the peripheral pool of splenic myeloid-derived suppressor cells (13), MIS416 treatment has been shown to induce innate IFN-γ, nitric oxide (NO), and IL-10 in healthy animal and human studies. As a result of the altered immune milieu, MIS416 treatment also promoted expansion of splenic regulatory T (Treg) cells, and in a model of neuroinflammation, this was associated with suppression of the inflammatory response mediated by T helper (Th) 1, Th2, and Th17 cells (14). As a result of its efficacy in experimental autoimmune encephalomyelitis, MIS416 is currently in clinical trials for multiple sclerosis (13, 14).

The mechanisms of action of MSCs in the therapeutic setting include both direct cell-to-cell contact as well as the secretion of soluble factors which modulate diverse immune cell subsets (15–18). hUCB-MSCs were detected in inflamed colons and alleviated the severity in mouse colitis model (5, 19), suggesting that their immunomodulatory effects occur in the localized environment, and that localization of MSCs at the inflammatory sites is a key factor for their therapeutic effects (20, 21). Accordingly, many studies have demonstrated the distribution of injected MSCs at the inflamed colon in experimental colitis model by various methods, such as luciferase, green fluorescent protein (GFP), or indocyanine green labeling (19, 22, 23). Several molecules, such as monocyte chemotactic protein-1 (MCP-1/CCL2), stromal cell-derived factor-1 (SDF-1/CXCL12), integrins, and matrix metalloproteinases, are involved in the recruitment of MSCs into inflamed tissues (24). Among these factors, MCP-1 is produced by various cells including monocytes/macrophages which are a major source of MCP-1 (25). It has also been reported that secreted MCP-1 stimulates migration of MSCs to the target region (26), and this has been demonstrated in an experimental rat model of stroke, where MCP-1 from ischemically damaged tissue was shown to facilitate migration of the transplanted human MSCs toward the site of injury (27).

As MIS416 co-administration might be a novel method for improving hUCB-MSC-based therapies against IBDs, in the present study, we investigated whether MIS416 co-administration could accelerate the therapeutic efficacy of hUCB-MSCs in a dextran sulfate sodium (DSS)-induced colitis model.

## Materials and Methods

### Mice

**Figure 1:**
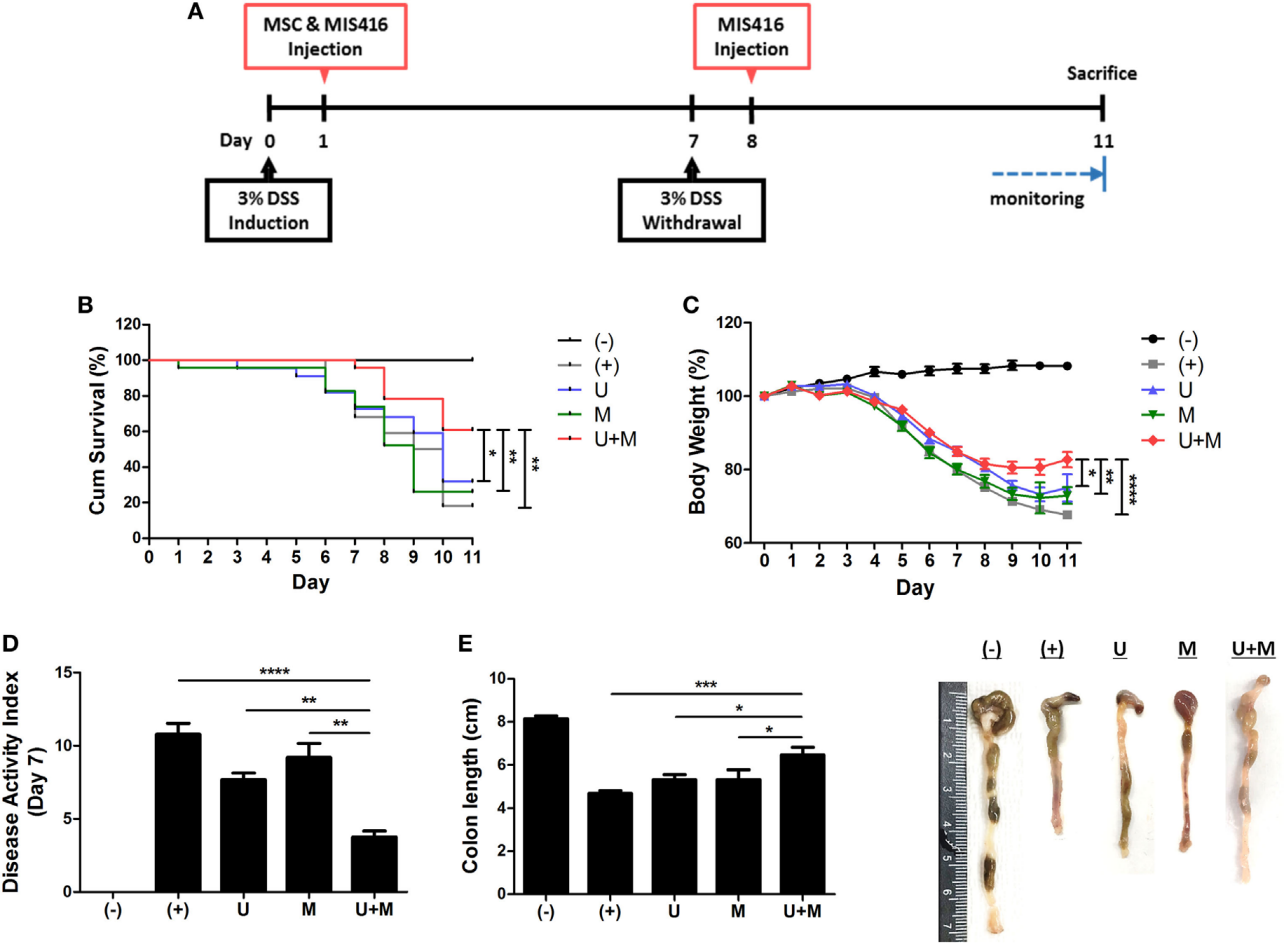
Simultaneous administration of MIS416 and human adult stem cells, including umbilical cord blood-derived mesenchymal stem cells (hUCB-MSCs) enhances therapeutic effects of the cells against experimental colitis. Mice were exposed to 3% dextran sulfate sodium (DSS) in their drinking water for 7 days and injected intraperitoneally hUCB-MSCs at day 1, and MIS416 at day 1 and 8 through retro-orbital route. **(A)** Scheme for the experimental design. **(B)** Survival rates of the mice were monitored. **(C)** Changes in body weights were measured daily. **(D)** Disease activity index for colitis severity. **(E)** On day 11, colon length of mice was measured by gross examination. *n* = 10–22 mice per group, three independent animal experiments were performed. (−): negative control group, (+): DSS administered group, U: hUCB-MSCs-treated group, M: MIS416-treated group, U + M: hUCB-MSCs and MIS416 co-treated group. *P < 0.05, **P < 0.01, ***P < 0.001, and ****P < 0.0001. Results are shown as the mean ± SEM.

All experimental processes were approved by the Seoul National University Institutional Animal Care and Use Committee (IACUC No. SNU-170523-3) in accordance with the guidelines of the committee. C57BL/6J mice (male, 6–8 weeks old) were obtained from Orientbio (Sungnam, Republic of Korea). Mice were housed in a temperature- and humidity-controlled room in the animal facility of Seoul National University. Colitis was experimentally induced by administration of 3% DSS (MP Biochemicals, Solon, OH, USA) in drinking water supplied *ad libitum* for 7 days unless the application of humane endpoint was needed, DSS treatment was replaced by normal drinking water after day 7. MIS416 (Innate Immunotherapeutics, Auckland, New Zealand) was injected into the retro-orbital sinuses on day 1 and day 8 as described in Figure 1A. Subsequently, hUCB-MSCs were suspended in phosphate-buffered saline (PBS) (2 × 10^6^ cells/200 μl per head) and infused into mice intraperitoneally on day 1. Body weight and survival rate were monitored over 12 days. On day 7, the therapeutic potential of the treatments was measured by evaluating the disease activity index (DAI), including body weight loss (0–4), stool consistency (0–4), bleeding (0–4), general activity (0–2), and coat roughness (0–4), with a maximum DAI score of 18 and the humane endpoint was established at DAI = 13.5. On day 11, colon, serum, and spleen samples were collected from sacrificed mice for further *ex vivo* examinations. To define the systemic influence of MIS416, mice were sacrificed a day after injection (day 2), and colon, serum, and spleen samples were collected for analyses.

### Histopathological Evaluation

The collected colon samples were fixed with 10% formalin. The tissues were embedded in paraffin, cut into 4-μm-thick sections, and stained with hematoxylin and eosin (H&E) and picrosirius red (PSR). The histopathological score was obtained by evaluating H&E stained slides based on the following five indexes: loss of goblet cells, infiltration of immune cells, crypt abscesses, hyperemia and edema, and loss of epithelium, with a maximum score of 15 (graded from 0–3 for the severity of each index). Generation of fibrotic tissue in the colon was assessed by PSR staining, followed by counterstaining with fast green (Sigma-Aldrich, St. Louis, MO, USA). The ratio of the fibrotic (PSR-positive) area was assessed using ImageJ software version 1.51j8 (National Institutes of Health, Bethesda, MD, USA).

### Cytokine Production

IL-6, IL-10, MCP-1, IFN-γ, TNF, and IL-12p70 in the serum of mice were measured by flow cytometry using the CBA Mouse Inflammation Kit (BD Bioscience, San Jose, CA, USA). IL-17A and IL-23 in the serum were determined by CBA mouse flex sets (BD Bioscience). IL-6 in hUCB-MSC-cultured medium was measured using the CBA Human Th1/Th2/Th17 Cytokines kit (BD Bioscience) according to the manufacturer’s instructions. Briefly, 50 µl of samples or standards were mixed with 50 µl of mixed capture beads and 50 µl of a Phycoerythrin (PE)-conjugated detection antibody and incubated for the designated time (2 h for mouse, 3 h for human samples). Detection was performed with a FACScalibur flow cytometer and evaluated using Cell Quest software (BD Bioscience). To address the level of neutrophil infiltration, myeloperoxidase (MPO) activity of colon samples was assessed using a Mouse Myeloperoxidase DuoSet kit (R&D Systems, Minneapolis, MN, USA) according to the manufacturer’s instructions. Briefly, colon segments were homogenized with the protein lysis buffer Pro-prep (Intron Biotechnology Co., Sungnam, Republic of Korea) to a concentration of 10 mg/ml. Appropriately diluted samples and standards were added to wells of 96-well plate pre-coated with a mouse MPO capture antibody and incubated overnight at room temperature. Then, a streptavidin–horseradish peroxidase (HRP)-conjugated detection antibody was added and incubated. After the incubation with streptavidin–HRP, the level of MPO was quantified by reaction with a substrate solution. Absorbance was read at 450 nm using an Infinite200 PRO microplate reader (Tecan, Maennedorf, Switzerland). After indicated treatment to hUCB-MSCs, the level of NO production was measured by Griess assay (Promega, Fitchburg, WI, USA). And the level of PGE_2_ secretion was determined by commercial ELISA kit (R&D Systems, Minneapolis, MN, USA). After enough reaction, the absorbance of the samples was measured at 540 and 450 nm, respectively, using an Infinite200 PRO microplate reader (Tecan).

### Isolation and Culture of hUCB-Derived Mononuclear Cells (hUCB-MNCs)

All experiments using human UCB or UCB-derived cells were approved by Institutional Review Board (IRB) of the Boramae Hospital and Seoul National University (IRB No. 1707/001-008) with informed maternal consent. Isolation was performed as previously described (28, 29). The UCB samples were mixed with HetaSep solution (Stem Cell Technologies, Vancouver, BC, Canada) at a ratio of 5:1. After incubation at room temperature for 1 h, the supernatant was carefully collected. Mononuclear cells were separated using Lymphoprep (Stem Cell Technologies) density gradient centrifugation. Isolated cells were seeded in growth medium consisting of RPMI 1640 (Gibco BRL, Grand Island, NY, USA) and 10% filtered fetal bovine serum (FBS), followed by washing with PBS. Isolated cells were cultured with 50 µg/ml of MIS416 for 3 days and used for further analyses.

### Isolation and Culture of hUCB-MSCs

Human adult stem cells, including hUCB-MSCs were isolated as previously described (30, 31). Isolated hUCB-MNCs were cultured with KSB-3 complete media (Kangstem Biotech, Seoul, Korea) containing 10% FBS (Gibco BRL) and antibiotics. After 3 days, non-adherent cells were removed, and adherent cell colonies were consistently cultured to establish sharp and spindle-shaped hUCB-MSCs. The cells possessed the characteristics of MSCs (30, 31) and were verified by expression of surface markers by flow cytometric analysis (Figure S1 in Supplementary Material). We used the cells at passage 8 for *in vivo* experiments and passage 8–10 for *in vitro* experiments.

### Cell Cycle Assay

After indicated treatment and harvest, hUCB-MSCs were washed in PBS twice prior to fixation with ice-cold 70% ethanol (over 30 min, −20°C). Fixed cells were washed in PBS and resuspended in 400 µl PBS, containing RNase A (6.25 µg/ml) and propidium iodide (50 µg/ml), and incubated at 37°C for 30 min. Cell cycle analysis was performed using a FACSCalibur flow cytometer and evaluated using Cell Quest software (BD Bioscience).

### Western Blot

Whole-cell lysates were prepared with the protein lysis buffer Pro-prep (Intron Biotechnology Co.), and the concentration was measured *via* the Bradford method using a Bio-Rad protein assay kit (Bio-Rad Laboratories, Hercules, CA, USA), with bovine serum albumin (BSA) as the standard. For each protein sample, a 10 µg aliquot was separated by 10% sodium dodecyl sulfate polyacrylamide gel electrophoresis and transferred to a nitrocellulose membrane. After blocking with 3% BSA in Tris-buffered saline, the blots were probed overnight at 4°C with the following primary antibodies: IDO-1 (BioLegend, San Diego, CA, USA), iNOS (Santa Cruz Biotechnology, Santa Cruz, CA, USA), COX-2 (Abcam, Cambridge, MA, USA), RIP2, MyD88 (Millipore, Billerica, MA, USA), IKK-α, IκB-α (Cell Signaling Technology, Beverly, MA, USA), phospho-NF-κB p65, total NF-κB p65 (Santa Cruz Biotechnology), phospho-JNK, total JNK, phospho-p38 mitogen-activated protein kinase (MAPK), total p38 MAPK, phospho-ERK, total ERK, and GAPDH (Cell Signaling Technology) as the housekeeping control. The membranes were blotted with secondary antibodies at room temperature for 1 h, and the proteins were detected with enhanced chemiluminescence reagent (GE Healthcare Life Science, Buckinghamshire, UK). Detailed information for all antibodies is provided in Table S1 in Supplementary Material.

### Cell Proliferation Assay

Proliferation of hUCB-MSCs and hUCB-MNCs was determined after treatment with MIS416. In addition, to identify the suppression ability of hUCB-MSCs, isolated CD4^+^ T cells were co-cultured with MIS416 pre-treated hUCB-MSCs, followed by a proliferation assay. Cell proliferation was measured using a bromodeoxyuridine (BrdU) ELISA kit (Roche, Indianapolis, IN, USA). After the indicated treatment, BrdU labeling solution (100 µM) was added into the culture medium in 96-well plate. The culture medium containing BrdU solution was removed after overnight incubation at 37°C, and the cells were fixed with FixDenat solution for 30 min at room temperature. The cells were then incubated with an anti-BrdU-POD working solution for 90 min at room temperature. Following three rinses with washing solution, a substrate solution was added to the cells and incubated for 5~30 min at room temperature. After sufficient color development, the OD values were read at 450 nm on a microplate reader (Tecan).

### Cell Migration Assay

Human adult stem cells, including hUCB-MSCs were suspended in culture medium, and 500 µl of the cell suspension (1 × 10^4^ cells/ml) was added to transwell inserts (8 µm pore size). Subsequently, 500 µl of MSC conditioned medium was added to the lower chambers. After 24 h of incubation, hUCB-MSCs that migrated to the underside of the membrane were fixed, and the remaining cells in the upper chamber were carefully swiped with a cotton swab. The membranes of the transwell were stained with DAPI and sealed on slides. A confocal microscope (Nikon, Eclipse TE200, Japan) was used to count the number of cells on the underside of the insert for each group.

### Flow Cytometry

To confirm the expression of cell surface markers, isolated primary cells were stained with a fluorochrome-conjugated antibody and analyzed. After cell surface staining, the cells were stained with antibodies against intracellular protein, as necessary. For intracellular staining, we used transcription factor buffer set (BD Biosciences, #562725) according to manufacturer’s instruction. Briefly, the cells were fixed by 1× Fix/Perm Buffer and permeabilized by 1× Perm/Wash Buffer. The following antibodies were used: fluorescein isothiocyanate (FITC)-conjugated anti-human CD4, allophycocyanin (APC)-conjugated anti-human IFN-γ, PE-conjugated anti-human IL-4, PE-conjugated anti-human FoxP3, PE-conjugated anti-human IL-17A, APC-conjugated anti-mouse CD4, PE-conjugated anti-mouse CD25, FITC-conjugated anti-mouse Foxp3, FITC-conjugated anti-human CD45, PE-conjugated anti-human HLA-DR, FITC-conjugated anti-human CD105 (Endoglin), APC-conjugated anti-human CD73, APC-conjugated anti-human CD29, FITC-conjugated anti-human CD44, PE-conjugated anti-human CD36, and PE-conjugated anti-human CD34. Detailed information for all antibodies is provided in Table S1 in Supplementary Material. Fluorescence was detected with a FACSCalibur flow cytometer and evaluated using Cell Quest software (BD Biosciences).

### Quantitative PCR

Total RNA was extracted from tissues and cultured cells by using TRIzol reagent (Invitrogen, Carlsbad, CA, USA). cDNA synthesis was performed using Superscript™ III reverse transcriptase (Invitrogen). Quantitative real-time PCR was performed using SYBR-Green PCR Master Mix with an ABI 7300 sequence detection system. The mRNA levels of each gene were normalized using GAPDH as the housekeeping gene.

### Immunohistochemistry

4-μm-thick paraffin-embedded sections of colon were deparaffinized and rehydrated. For permeabilization, the samples were incubated with 0.05% Triton X-100 solution at room temperature for 10 min and blocked with 5% normal goat serum at room temperature for 1 h. Then, the cells were stained with rat anti-mouse Foxp3 monoclonal antibody (eBioscience, San Diego, CA, USA). Before imaging, nuclei were counterstained with DAPI. The images were captured by a confocal microscope (Nikon). Detailed information for all antibodies is provided in Table S1 in Supplementary Material.

### *In Vivo* Cell Tracking

For *in vivo* cell trafficking, hUCB-MSCs were transduced with a GFP-encoding retroviral vector. For retrovirus preparation, the pMX-GFP vector and retrovirus packaging vectors were co-transfected into 293FT cell (Invitrogen) using FuGENE 6 transfection reagent (Promega). Viral supernatants were collected at 48 h post-transfection, filtered through a 0.45 µm PVDF membrane filter, and then directly used to infect hUCB-MSCs. Transduction efficiency was monitored by fluorescence microscopy and flow cytometric analysis (Figure S2 in Supplementary Material). These cells (2 × 10^6^ cells/head) were intraperitoneally injected into mice on day 1. On day 2 and day 11, mice were sacrificed, and colon samples were collected. The distribution of hUCB-MSCs was imaged by confocal imaging (Nikon, Eclipse TE200, Japan) using 4-μm-thick paraffin-embedded sections. Further, colon samples were chopped and filtered to obtain a single-cell suspension, on day 2 and day 11. Then, samples were stained with antibody of hUCB-MSCs surface markers, anti-human CD29, and fluorescence was detected by flow cytometric analysis.

### Statistical Analysis

The mean values of all data are expressed as the mean ± SEM. Statistical analysis was performed using GraphPad Prism version 7.0 (GraphPad Software, San Diego, CA, USA). Data were assessed for normality using the D’Agostino and Pearson normality test. Where data were normally distributed, the significance of the data was determined by using Student’s *t*-tests for comparison of two groups or one-way ANOVA coupled to Bonferroni’s test for multiple groups. Where data were not normally distributed, Mann–Whitney *U* test or Kruskal–Wallis method coupled to Dunn’s multiple comparison test were used. For analysis of survival data, Kaplan–Meier test and Mantel–Cox post-test were used. Nonparametric tests were conducted by Kruskal–Wallis method coupled to Dunn’s multiple comparison test. *P*-values less than 0.05 were considered to be statistically significant and are indicated in the text.

## Results

### MIS416 Enhances the Therapeutic Effect of hUCB-MSCs Against DSS-Induced Colitis in Mice

The ligands of pattern-recognition receptors, as components of innate immune systems, affect the diverse functions of hUCB-MSCs, including immunomodulation, migration, proliferation, differentiation, and cytokine secretion (16, 32–35). To investigate whether MIS416, a microparticle comprising NOD2 and TLR9 agonists, could enhance the therapeutic effects of hUCB-MSCs, we co-injected hUCB-MSCs and MIS416 in DSS-induced colitis mice. The survival rate was further increased by co-treatment compared to hUCB-MSC or MIS416 single treatments (Figure 1B) [(+) vs U + M *p* = 0.003; U vs U + M *p* = 0.04; M vs U + M *p* = 0.006]. The therapeutic effects were also determined by attenuation of body weight loss—the body weight in the co-treatment group was markedly increased compared with hUCB-MSC- or MIS416-treated groups (Figure 1C) [(+) vs U + M *p* < 0.0001; U vs U + M *p* = 0.0444; M vs U + M *p* = 0.0050]. By assessment of DAI on day 7, it was confirmed that the severity of DSS-induced colitis was further attenuated by co-administration of MIS416 and hUCB-MSCs compared to each single treatment (Figure 1D) [(+) vs U + M *p* < 0.0001; U vs U + M *p* = 0.0025; M vs U + M *p* = 0.0025]. We next measured the length of colon on day 11, and the colon lengths were significantly increased in co-treated group compared to each single treated groups (Figure 1E) [(+) vs U + M *p* = 0.0003; U vs U + M *p* = 0.0294; M vs U + M *p* = 0.0275]. Taken together, these results suggest that co-treatment with MIS416 enhances the therapeutic efficacy of hUCB-MSCs against DSS-induced colitis.

### MIS416 Improves the Anti-Inflammatory Function and Tissue Regenerative Capacity of hUCB-MSCs in the Colon

**Figure 2:**
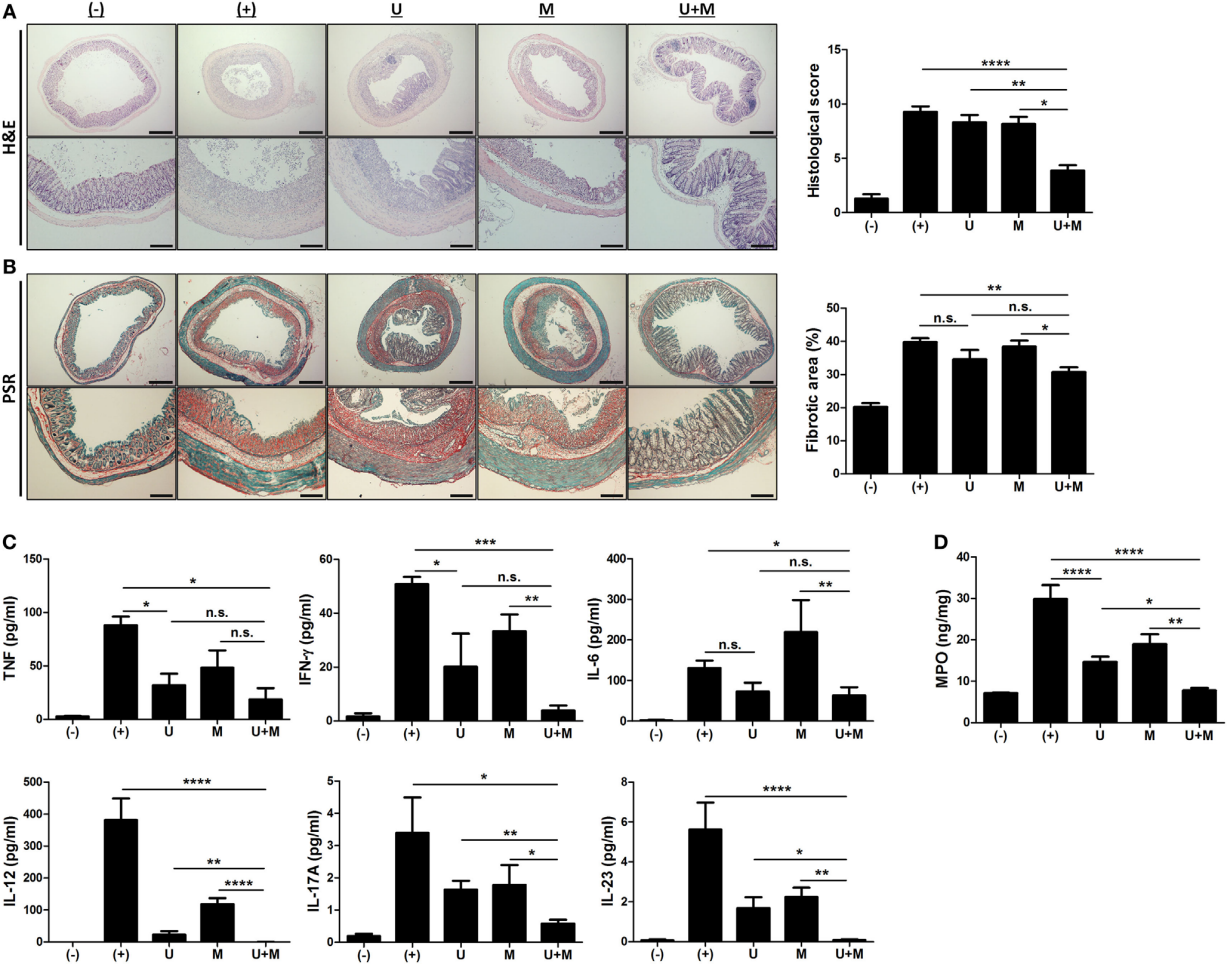
Co-administration of MIS416 and human adult stem cells, including umbilical cord blood-derived mesenchymal stem cells (hUCB-MSCs) attenuates inflammation and fibrosis in mice **(A)** Left: representative images of hematoxylin and eosin-stained sections, bar = 500 µm (upper), 200 µm (lower). Right: histopathological scoring was performed based on the evaluation criteria. **(B)** Left: representative images of picrosirius red-stained sections, bar = 500 µm (upper), 200 µm (lower). Right: the fibrotic areas were quantified. **(C)** The levels of tumor necrosis factor (TNF), interferon (INF)-γ, IL-6, IL-12, IL-17A, and IL-23 in the serum of mice were evaluated on day 11 by CBA. **(D)** Myeloperoxidase (MPO) activity of colon was measured as an indicator for neutrophil infiltration. *n* = 10–22 mice per group, three independent animal experiments were performed. (−): negative control group, (+): DSS administered group, U: hUCB-MSCs-treated group, M: MIS416-treated group, U + M: hUCB-MSCs and MIS416 co-treated group. *P < 0.05, **P < 0.01, ***P < 0.001, and ****P < 0.0001. Results are shown as the mean ± SEM.

We next examined H&E-stained colon samples to investigate the histopathological changes in the colons of DSS colitis mice on day 11. In the same context as previous results, colonic inflammation was more effectively resolved by co-treatment with MIS416 and hUCB-MSCs than each single treatment (Figure 2A) [(+) vs U + M *p* < 0.0001; U vs U + M *p* = 0.0051; M vs U + M *p* = 0.0103]. In addition, fibrosis-associated mucosal and submucosal collagen depositions were quantified by PSR staining. Treatment of hUCB-MSCs or MIS416 alone did not show significant changes, on the other hand, only co-treatment markedly decreased fibrosis and enhanced tissue regeneration (Figure 2B) [(+) vs U *p* = 0.2537; (+) vs U + M *p* = 0.0011; U vs U + M *p* > 0.9999; M vs U + M *p* = 0.0254]. Levels of proinflammatory cytokines, such as TNF, IFN-γ, IL-6, IL-12, IL-17A, and IL-23 were elevated in the serum of DSS-induced colitis mice. The levels of TNF were decreased in both hUCB-MSC- and co-treated mice serum, but significant change was not detected between two groups [(+) vs U *p* = 0.0234; (+) vs U + M *p* = 0.0177; U vs U + M *p* > 0.9999; M vs U + M *p* > 0.9999]. With IFN-γ and IL-6, although significant change was not detected between hUCB-MSC treatment and co-treatment, mice administered both MIS416 and hUCB-MSCs showed a greater significant decrease compared to positive control group [for IFN-γ, (+) vs U *p* = 0.0401; (+) vs U + M *p* = 0.0003; U vs U + M *p* = 0.5635; M vs U + M *p* = 0.0034/for IL-6, (+) vs U *p* > 0.9999; (+) vs U + M *p* = 0.0245; U vs U + M *p* > 0.9999; M vs U + M *p* = 0.0082]. The levels of IL-12, IL-17A, and IL-23 were also further decreased in the co-administration group compared with each single treatment groups, respectively (Figure 2C) [for IL-12, (+) vs U + M *p* < 0.0001; U vs U + M *p* = 0.0066; M vs U + M *p* < 0.0001/for IL-17A, (+) vs U + M *p* = 0.0119; U vs U + M *p* = 0.0019; M vs U + M *p* = 0.0483/for IL-23, (+) vs U + M *p* < 0.0001; U vs U + M *p* = 0.0192; M vs U + M *p* = 0.0086]. We next measured MPO activity to evaluate the infiltration of immune cells, particularly neutrophils. DSS-induced increases in MPO activity were rescued by injection of hUCB-MSCs, and co-administration with MIS416 significantly improved this rescue (Figure 2D) [(+) vs U *p* < 0.0001; (+) vs U + M *p* < 0.0001; U vs U + M *p* = 0.0172; M vs U + M *p* = 0.0011]. These findings demonstrate that MIS416 facilitates hUCB-MSCs to reduce secretion of proinflammatory cytokines and diminish tissue degeneration more effectively in the DSS-induced colitis model.

### MIS416 Does Not Directly Affect the Proliferation and Immunomodulatory Functions of hUCB-MSCs

**Figure 3:**
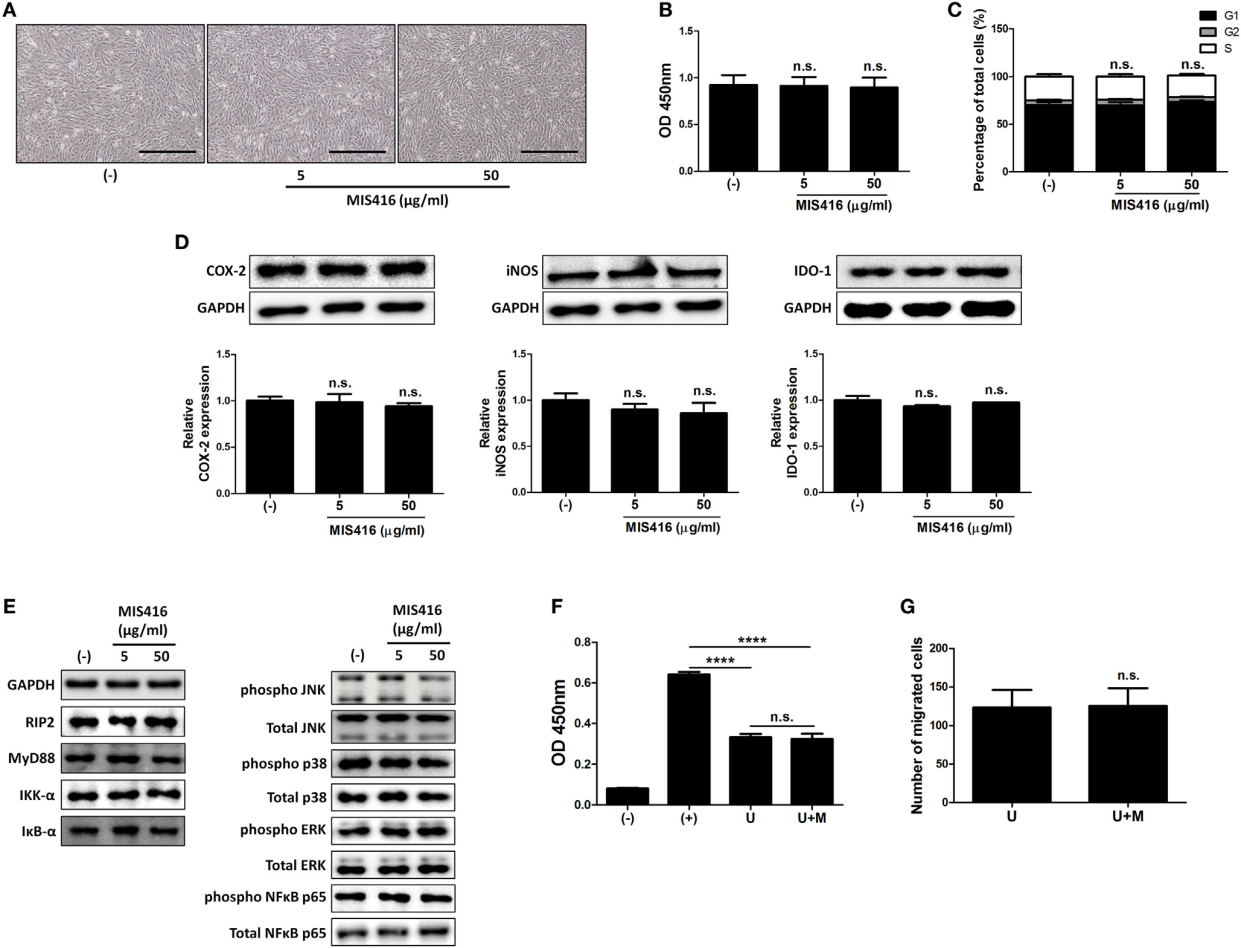
MIS416 does not have direct effect on the properties of human adult stem cells, including umbilical cord blood-derived mesenchymal stem cells (hUCB-MSCs) including immunomodulatory functions *in vitro*. **(A–E)** hUCB-MSCs were treated with indicated concentrations of MIS416 for 24 h and further analyses were conducted. **(A)** Representative of bright-field microscopy images of hUCB-MSCs, bar = 500 µm. **(B)** Proliferation of hUCB-MSCs was determined by BrdU ELISA kit. **(C)** Cell cycle assay. **(D)** The relative expression levels of COX2, iNOS, and IDO-1 in hUCB-MSCs were analyzed by immunoblotting analysis. **(E)** (Left) Expression levels of RIP2, MyD88, IKK-α, and IκB-α/(Right) phosphorylation levels of NFκB p65, p38 mitogen-activated protein kinase, ERK, and JNK were determined by western blot analysis. **(F)** Mixed lymphocytes reaction assay of hUCB-MSCs. **(G)** Transwell migration assay was performed and migrated cells were quantified. Gel electrophoresis was conducted under the same experimental conditions, and images of blots were cropped. Uncropped blot images are shown in Figure S7 in Supplementary Material. Experiments were performed in triplicate. (−): negative control group, (+): ConA activated group, U: hUCB-MSCs, U + M: MIS416-treated hUCB-MSCs. ****P < 0.0001. Results are shown as the mean ± SEM.

We next investigated whether MIS416 priming could affect the therapeutic properties of hUCB-MSCs. hUCB-MSCs were treated with the indicated concentrations of MIS416. MIS416 treatment did not cause any significant change in the morphology of the cells (Figure 3A). To investigate alterations in the proliferative capacity, the BrdU assay was performed. MIS416 treatment did not change the proliferation of hUCB-MSCs (Figure 3B) [(−) vs MIS416 5 µg/ml *p* = 0.9978; (−) vs MIS416 50 µg/ml *p* = 0.9807]. In the same context with the proliferation, MIS416 administration did not influence on the cell cycle profile of hUCB-MSCs (Figure 3C) [(−) vs MIS416 5 µg/ml *p* > 0.9999; (−) vs MIS416 50 µg/ml *p* = 0.9469].

To examine whether MIS416 could regulate the immunomodulatory ability of hUCB-MSCs, we evaluated the expression levels of COX-2, iNOS, and IDO-1, which are enzymes related to immunomodulation by hMSCs (36–38). Western blot analysis revealed that MIS416 treatment had no significant effect on the expressions of COX-2, iNOS, and IDO-1 (Figure 3D) [for COX-2, (−) vs MIS416 5 µg/ml *p* = 0.9817; (−) vs MIS416 50 µg/ml *p* = 0.7894/for iNOS, (−) vs MIS416 5 µg/ml *p* = 0.7031; (−) vs MIS416 50 µg/ml *p* = 0.5383/for IDO-1, (−) vs MIS416 5 µg/ml *p* = 0.3366; (−) vs MIS416 50 µg/ml *p* = 0.7858]. Consistently, levels of PGE_2_ and NO secretion into the culture medium were not altered by MIS416 priming (Figure S3A in Supplementary Material) [for PGE_2_, (−) vs MIS416 50 µg/ml *p* = 0.7294/for NO, (−) vs MIS416 50 µg/ml *p* = 0.7412]. The level of IL-6 secretion also did not exhibit any significant change (Figure S3A in Supplementary Material) [(−) vs MIS416 50 µg/ml *p* = 0.7679]. In addition, key downstream adaptor molecules, RIP2 for NOD2 and MyD88 for TLR9 were not changed by MIS416 treatment. The phosphorylation levels of signaling cascades, NF-κB and MAPK were also not altered (Figure 3E; Figure S3B in Supplementary Material). Consistent with previous studies (5, 30), the proliferation of hMNCs co-cultured with hUCB-MSCs was markedly inhibited. However, hUCB-MSCs primed with MIS416 did not show a significant difference from unstimulated cells (Figure 3F) [(+) vs U *p* < 0.0001; (+) vs U + M *p* < 0.0001; U vs U + M *p* > 0.9999]. We next evaluated the migratory ability of hUCB-MSCs primed with MIS416 *in vitro*. The number of migrated cells remained unchanged after MIS416 treatment (Figure 3G) (U vs U + M *p* = 0.9489). Overall, these findings suggest that MIS416 augments the therapeutic abilities of hUCB-MSCs by indirect mechanism, but not by direct influence on the cells.

### Exposure to MIS416 Causes Increases in the Number of Immune Cells *via* Activation of Innate Immune Cells Such as CD14^+^ Macrophages

**Figure 4:**
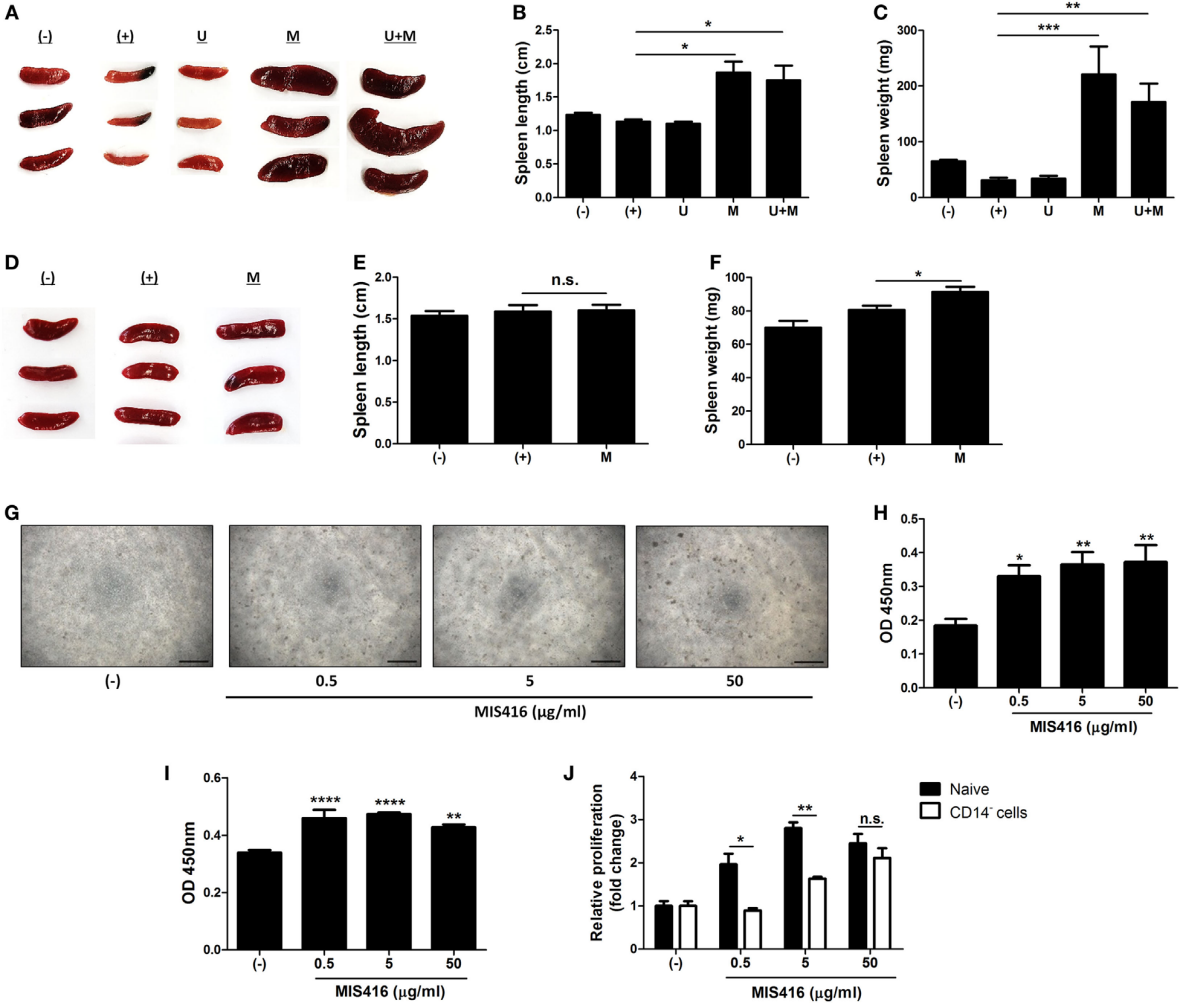
Injection of MIS416 alters systemic immune milieu of dextran sulfate sodium (DSS) colitis mice. **(A–C)** Each group of mice were sacrificed on day 11. **(A)** Gross examination of spleen. **(B)** Length and **(C)** weight of spleens were measured. **(D–F)** Each group of mice were sacrificed a day after MIS416 administration (day 2). **(D)** Gross examination of spleen. **(E)** Length and **(F)** weight of spleens were measured. **(G,H)** Human umbilical cord blood-derived mononuclear cells were cultured with MIS416 for 3 days. **(G)** Representative of bright-field microscopy images of hUCB-MNCs, bar = 100 µm. **(H)** Proliferation of hUCB-MNCs was determined by bromodeoxyuridine (BrdU) assay. **(I,J)** hUCB-MNCs or -derived immune cells were treated with indicated concentrations of MIS416 for 3 days. Proliferation of **(I)** isolated CD14^+^ macrophage-like cells and relative proliferations of **(J)** naive and CD14^+^ cell-depleted hUCB-MNCs and were measured by BrdU ELISA kit. *n* = 5–9 mice per group, two independent animal experiments were performed. *In vitro* experiments were performed in triplicate. (−): Negative control group, (+): DSS administered group, U: hUCB-MSCs treated group, M: MIS416-treated group, U + M: hUCB-MSCs and MIS416 co-treated group. *P < 0.05, **P < 0.01, ***P < 0.001, ****P < 0.0001. Results are shown as the mean ± SEM.

We observed that co-treatment with hUCB-MSCs and MIS416 was better than either treatment alone in DSS-induced colitis, although MIS416 had no direct effect on hUCB-MSCs. Thus, we hypothesized that MIS416 would indirectly upregulate the therapeutic effects of hUCB-MSCs by targeting NOD2 or TLR9 on other cells. We investigated changes in the spleen, the largest secondary lymphoid organ which contains various immune cells, after systemic administration of MIS416 on day 11. Significant enlargement of the spleen was identified in both mice treated with MIS416 alone and co-treated with hUCB-MSCs (Figure 4A). The length and weight of the spleen were considerably increased in MIS416-treated groups (Figures 4B,C) [for length, (+) vs M *p* = 0.0109; (+) vs U + M *p* = 0.0254/for weight, (+) vs M *p* = 0.0002; (+) vs U + M *p* = 0.0049]. To determine whether MIS416 elicited a detectable splenic response at an earlier time point, we investigated the spleen of mice a day after MIS416 treatment (on day 2). Although there was no significant change in the length (Figures 4D,E), the weight of the spleen was increased (Figure 4F) [for length, (+) vs M *p* > 0.9999/for weight, (+) vs M *p* = 0.0458]. We mimicked this phenomenon *in vitro* by treating hUCB-MNCs with MIS416 and observed that cell proliferation was dose-dependently elevated (Figures 4G,H) [(−) vs MIS416 0.5 µg/ml *p* = 0.0419; (−) vs MIS416 5 µg/ml *p* = 0.0062; (−) vs MIS416 50 µg/ml *p* = 0.0041]. Furthermore, isolated CD4^+^ T cells did not proliferate in the presence of MIS416 (Figure S4A in Supplementary Material). By contrast, proliferation of CD14^+^ cells was increased in response to MIS416 (Figure 4I) [(−) vs MIS416 0.5 µg/ml *p* < 0.0001; (−) vs MIS416 5 µg/ml *p* < 0.0001; (−) vs MIS416 50 µg/ml *p* = 0.0035], and depletion of CD14^+^ cells led to decreased proliferation of hUCB-MNCs (Figure 4J) (Naive vs CD14^−^ cells, at MIS416 0.5 µg/ml *p* = 0.0131; at MIS416 5 µg/ml *p* = 0.0011; at 50 µg/ml *p* = 0.3409). In addition, MIS416 increased or maintained the expression of M1-type genes, but suppressed M2-type features of CD14^+^ cells (Figure S4B in Supplementary Material). These findings suggest that MIS416 systemically stimulates the immune environment and increases the number of activated immune cells through activation of innate immune cells, such as CD14^+^ macrophages.

### MIS416 and hUCB-MSCs Collaborate in the Modulation of Intestinal Immune Balance by Regulating Polarization of Th Cell Lineages

**Figure 5:**
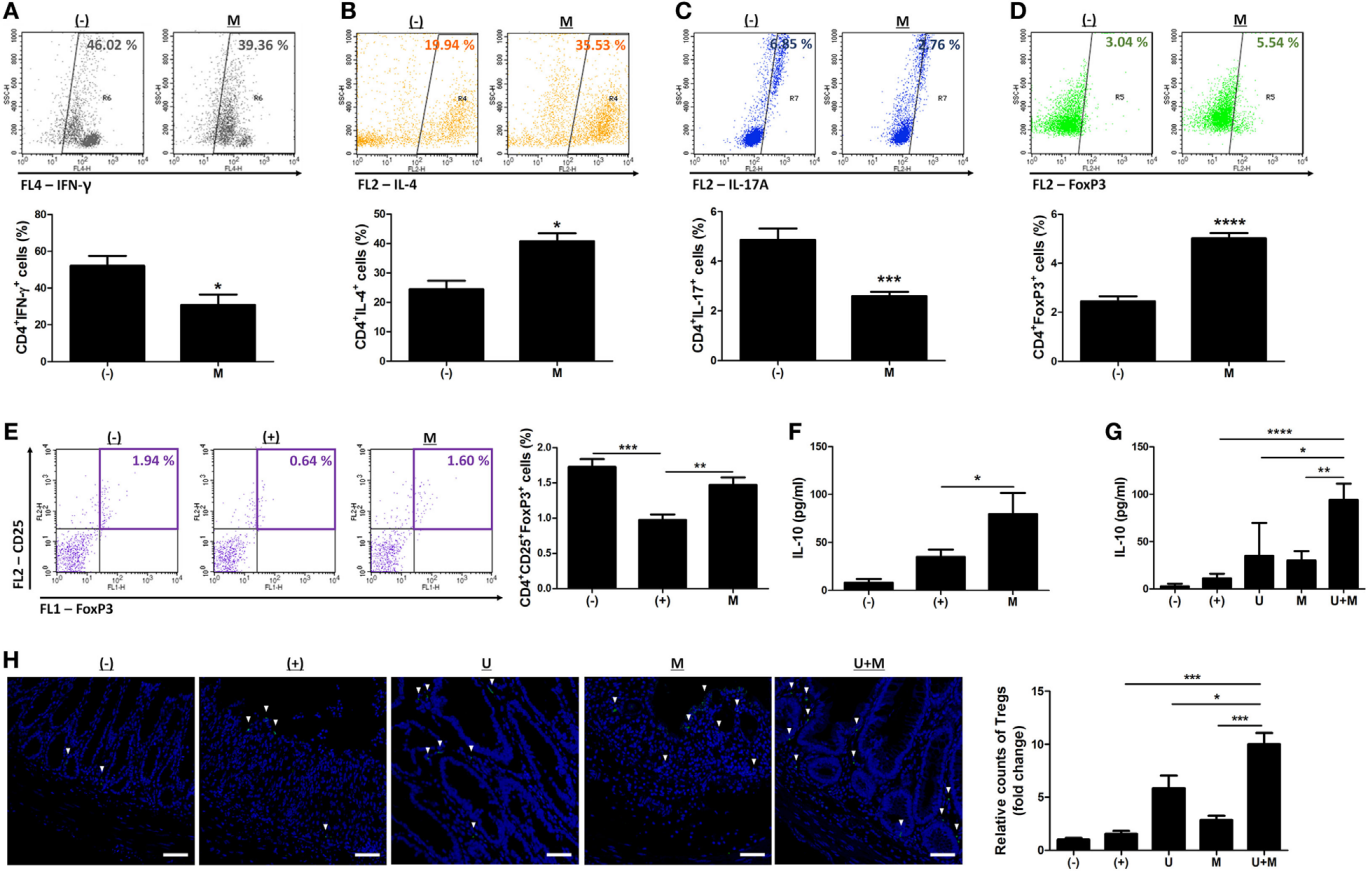
MIS416 and human adult stem cells, including umbilical cord blood-derived mesenchymal stem cells (hUCB-MSCs) cooperate in intestinal homeostasis by regulating polarization of T helper (Th) cells. **(A–D)** hUCB-MNCs were cultured with MIS416 for 3 days and analyzed for each population of Th lineages by flow cytometric analysis. Populations of **(A)** CD4^+^IFN (interferon)-γ^+^ cells for Th1, **(B)** CD4^+^IL-4^+^ cells for Th2, **(C)** CD4^+^IL-17A^+^ cells for Th17, and **(D)** CD4^+^FoxP3^+^ cells for regulatory T cells were determined. **(E,F)** Each group of mice were sacrificed a day after MIS416 administration (day 2). **(E)** Populations of CD4^+^CD25^+^FoxP3^+^ cells in spleen were analyzed by flow cytometric analysis. **(F)** The levels of IL-10 in serum were detected by CBA analysis. **(G,H)** Each group of mice was sacrificed on day 11. **(F)** The levels of IL-10 in serum were measured by CBA. **(G)** Representative images of FoxP3-immunostained colons, bar = 100 µm, and the quantification. ▾; FoxP3^+^ cells. *n* = 7–8 mice per group, two independent animal experiments were performed. *In vitro* experiments were performed in triplicate. (−): negative control group, (+): DSS administered group, U: hUCB-MSCs-treated group, M: MIS416-treated group, U + M: hUCB-MSCs and MIS416 co-treated group. *P < 0.05, **P < 0.01, ***P < 0.001, ****P < 0.0001. Results are shown as the mean ± SEM.

We further investigated whether MIS416 treatment altered the proportions of immune cells in hUCB-MNCs using flow cytometric analysis. Consistent with previous studies (7, 14), the proportion of proinflammatory effector cells, including CD3^+^, CD4^+^, and CD8^+^ cells, were decreased whereas CD19^+^ cells were increased (Figure S5 in Supplementary Material) [for CD3^+^ cells, (−) vs M *p* = 0.0023/for CD4^+^ cells, (−) vs M *p* < 0.0001/for CD8^+^ cells, (−) vs M *p* = 0.0021/for CD19^+^ cells, (−) vs M *p* = 0.0142]. We further explored the systemic effects of MIS416 on cell fate decisions in the Th cell lineages. Treatment with MIS416 reduced the populations of CD4^+^IFN-γ^+^ Th1 cells (Figure 5A) and increased the proportions of CD4^+^IL-4^+^ Th2 cells in hUCB-MNCs (Figure 5B) [for CD4^+^IFN-γ^+^ Th1 cells, (−) vs M *p* = 0.0354/for CD4^+^IL-4^+^ Th2 cells, (−) vs M *p* = 0.0142]. The proportion of CD4^+^IL-17A^+^ cells was decreased in the presence of MIS416 (Figure 5C) [(−) vs M *p* = 0.0002]. In addition, we determined that treatment with MIS416 augmented the proportion of CD4^+^FoxP3^+^ Treg cells in hUCB-MNCs (Figure 5D) [(−) vs M *p* < 0.0001]. We also confirmed increased Treg cells *in vivo*, detecting higher numbers of CD4^+^CD25^+^FoxP3^+^ Treg cells in the colons of MIS416-treated mice than DSS-induced mice on day 2 (Figure 5E) [(+) vs M *p* = 0.0050]. We next determined the level of IL-10, which can act as both an inducer and effector cytokine of Treg cells. On day 2, the expression level of IL-10 was altered by MIS416 injection (Figure 5F) [(+) vs M *p* = 0.0131]. By day 11, MIS416-treated mice showed slightly increased levels of IL-10, whereas robust serum IL-10 expression was identified in mice co-treated with MIS416 and hUCB-MSCs (Figure 5G) [(+) vs U + M *p* < 0.0001; U vs U + M *p* = 0.0284; M vs U + M *p* = 0.0069]. In the same context, the colonic infiltration of Foxp3^+^ Treg cells was significantly increased in the colons of mice treated with MIS416 and hUCB-MSCs than with either MIS416 or hUCB-MSCs alone (Figure 5H) [(+) vs U + M *p* = 0.0001; U vs U + M *p* = 0.0313; M vs U + M *p* = 0.0006]. These findings indicate that MIS416 alters the immune cell composition by suppressing effector cells and promoting regulatory cells, which are remarkably augmented by co-administration of MIS416 and hUCB-MSCs.

### Alteration of the Immune Environment by MIS416 Improves the Immunosuppressive Effect of hUCB-MSCs

**Figure 6:**
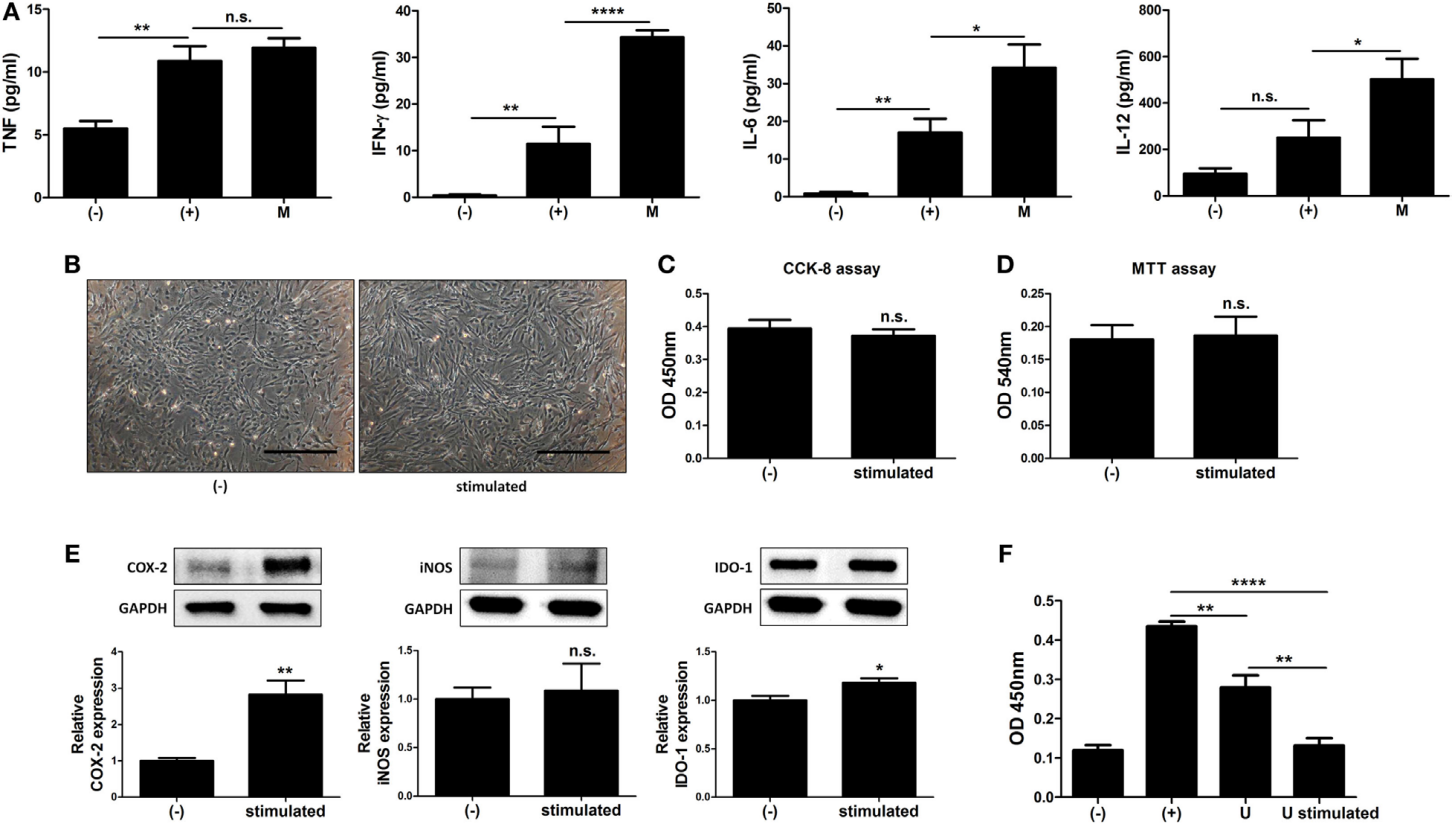
MIS416-mediated increase of cytokines upregulate immunomodulative function of human adult stem cells, including umbilical cord blood-derived mesenchymal stem cells (hUCB-MSCs). **(A)** The levels of tumor necrosis factor (TNF), interferon (IFN)-γ, IL-6, and IL-12 in serum of mice were evaluated a day after MIS416 administration (day 2) by CBA analysis. **(B–F)** Combination of cytokines, IL-6 (25 ng/ml), IL-12 (20 ng/ml), and IFN-γ (20 ng/ml), were used to treat hUCB-MSCs for 24 h. **(B)** Representative of bright-field microscopy images of hUCB-MSCs, bar = 500 µm. Proliferation and cell viability of hUCB-MSCs were determined by **(C)** CCK-8 assay and **(D)** MTT assay. **(E)** The expression levels of COX2, iNOS, and IDO-1 in hUCB-MSCs were analyzed using western blot analysis. **(F)** hUCB-MNCs were co-cultured with hUCB-MSCs stimulated with the cytokines directly and their proliferation was determined by bromodeoxyuridine ELISA assay. *n* = 7–8 mice per group, two independent animal experiments were performed. *In vitro* experiments were performed in triplicate. Gel electrophoresis was conducted under the same experimental conditions, and images of blots were cropped. Uncropped blot images are shown in Figure S7 in Supplementary Material. (−): negative control group, (+): DSS administered group in **(A)** and ConA activated group in **(F)**, M: MIS416-treated group, U: hUCB-MSCs, stimulated/U stimulated: cytokine cocktail-treated hUCB-MSCs. *P < 0.05, **P < 0.01, ****P < 0.0001. Results are shown as the mean ± SEM.

The immune system in the peritoneal cavity dynamically interacts with both the lymphatic system and general circulation by exchange of the fluids and cells, and intraperitoneally infused hMSCs remain in peritoneal cavity while interacting with the peritoneal immune system (39, 40). To address whether MIS416-mediated immune change affected immunomodulation of hUCB-MSCs, we analyzed the cytokine profiles in the serum of MIS416-treated mice on day 2. Although the level of TNF was not significantly elevated, it was observed that secretion of IFN-γ, IL-6, and IL-12 was markedly increased by MIS416 infusion on day 2 (Figure 6A) [for TNF, (+) vs M *p* > 0.9999/for IFN-γ, (+) vs M *p* < 0.0001/for IL-6, (+) vs M *p* = 0.0311/for IL-12, (+) vs M *p* = 0.0354]. Based on these results, MIS416-mediated immune cell stimulation altered secretory profile of cytokines in the serum of mice. These cytokines are reported to activate the immunomodulatory functions of MSCs (6, 41–44). To investigate whether these cytokines could promote therapeutic potential of hUCB-MSCs, the cells were cultured with IL-6, IL-12, and IFN-γ for 24 h. Combination of these cytokines did not show any effects on the proliferation or viability of hUCB-MSCs (Figures 6B–D) [for CCK-8 assay, (−) vs stimulated *p* = 0.5121/for MTT assay, (−) vs stimulated *p* = 0.8722]. However, expression levels of COX-2 and IDO-1 were increased by treatment of cytokine cocktail; by contrast, iNOS expression was not changed (Figure 6E) [for COX-2, (−) vs stimulated *p* = 0.0099/for iNOS, (−) vs stimulated *p* = 0.7819/for IDO-1, (−) vs stimulated *p* = 0.0494]. Consistently, secretion of PGE_2_ was increased, whereas the level of NO in the culture medium was not altered by cytokine cocktail (Figure S6 in Supplementary Material) [for PGE_2_, (−) vs stimulated *p* = 0.0022/for NO, (−) vs stimulated *p* = 0.100]. To define the inhibitory effects of stimulated hUCB-MSCs on immune cells, proliferation of hUCB-MNCs was analyzed. As a result, proliferation of hUCB-MNCs was shown to be decreased by hUCB-MSCs (Figure 6F) [(+) vs U *p* = 0.0071]. Importantly, the suppression was enhanced by treatment with a cytokine cocktail (Figure 6F) [(+) vs U stimulated *p* < 0.0001; U vs U stimulated *p* = 0.0020]. Taken together, we have demonstrated that MIS416-mediated increase of secreted cytokines, such as IL-6, IL-12, and IFN-γ enhance the immunosuppression of hUCB-MSCs.

### MIS416-Induced Secretion of MCP-1 Promotes the Migration of hUCB-MSCs

**Figure 7:**
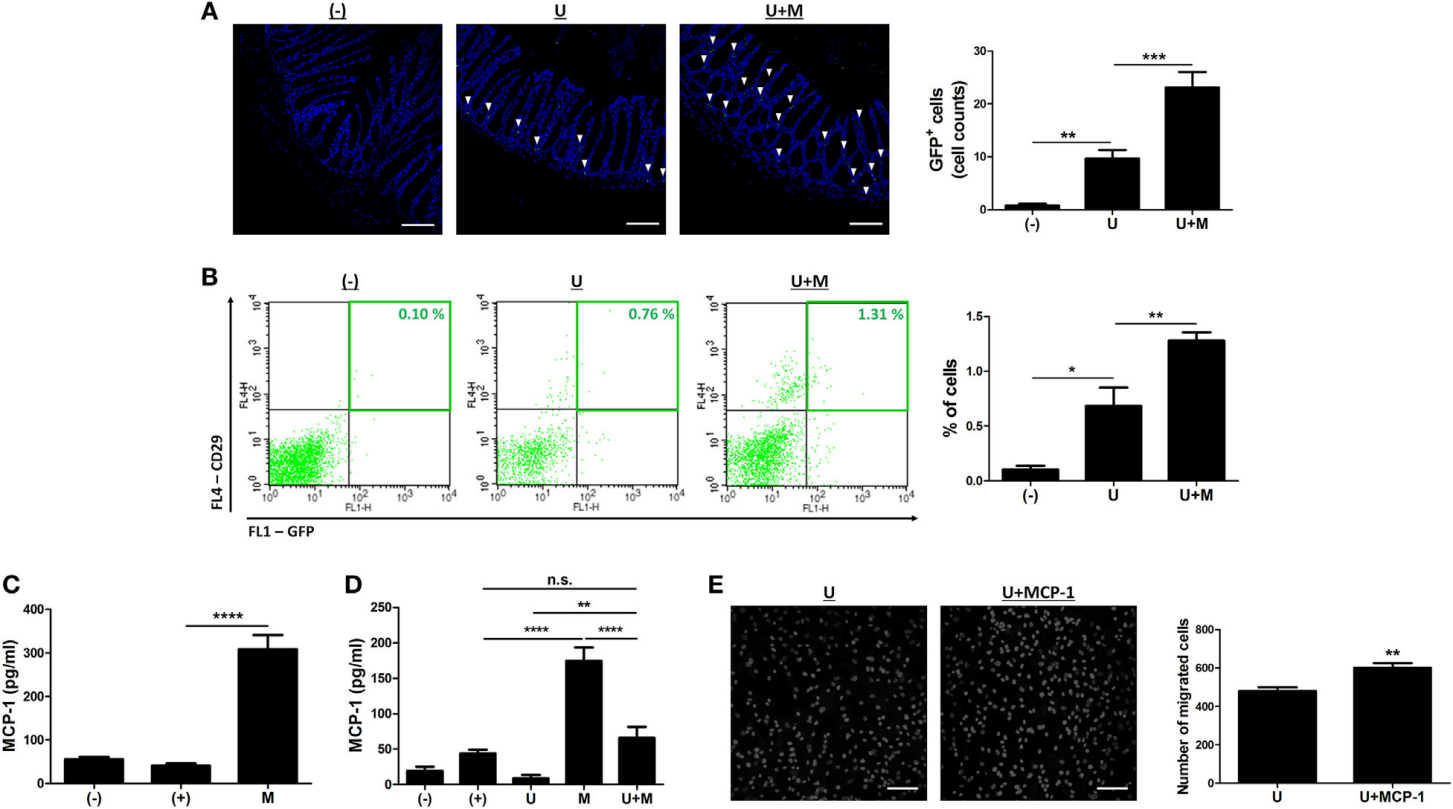
MIS416 increases the systemic level of MCP-1 which promotes migration of human adult stem cells, including umbilical cord blood-derived mesenchymal stem cells (hUCB-MSCs) toward the lesion. **(A,B)** Green fluorescent protein (GFP) expressing hUCB-MSCs were injected intraperitoneally into dextran sulfate sodium (DSS)-induced colitis mice. The colon sections were examined for green fluorescent cells using a confocal microscope and flow cytometer a day after injection (day 2). **(A)** Representative images of colons by confocal microscopy, bar = 100 µm, and the quantification. ▾; eGFP positive cells. **(B)** Populations of GFP positive hUCB-MSCs were analyzed by flow cytometric analysis. **(C,D)** Levels of MCP-1 in serum were evaluated by CBA. Each group of mice was sacrificed on **(C)** day 2 and **(D)** day 11. **(E)** hUCB-MSCs were incubated with MIS416 for 24 h. *In vitro* migration ability of hUCB-MSCs was determined using Transwell migration assay. Representative of confocal microscopy images of migrated hUCB-MSCs, bar = 100 µm, and the quantification. *n* = 8–11 mice per group, three independent animal experiments were performed. *In vitro* experiments were performed in triplicate. (−): negative control group, (+): DSS administered group, U: hUCB-MSCs treated group, M: MIS416-treated group, U + M: hUCB-MSCs and MIS416 co-treated group. *P < 0.05, **P < 0.01, ***P < 0.001, ****P < 0.0001. Results are shown as the mean ± SEM.

The engraftment to inflamed sites is crucial for therapeutic potential of hUCB-MSCs (19, 22, 23). Therefore, we investigated whether MIS416 administration could upregulate the mobilization of hUCB-MSCs into inflamed colon. DSS colitis mice were intraperitoneally injected with GFP positive hUCB-MSCs in the presence or absence of MIS416. On day 2, GFP positive hUCB-MSCs were detected in inflamed colon by immunohistochemistry (Figure 7A) [(−) vs U *p* = 0.0083]. hUCB-MSCs were more frequently detected in colon of co-treated mice than hUCB-MSCs treated mice (Figure 7A) [U vs U + M *p* = 0.0001]. However, hUCB-MSCs were not detected in the colon of the mice on day 11 (data not shown), which was also confirmed by flow cytometric analysis (Figure 7B) [(−) vs U *p* = 0.0207; U vs U + M *p* = 0.0083]. We next investigated which molecules affected the migratory ability of hUCB-MSCs in the presence of MIS416. MCP-1 is known to stimulate migration of MSCs to the target region (26). To confirm whether MIS416 caused an increase of MCP-1, the sera of each group were collected on day 2. As a result, the mice injected with MIS416 showed markedly increased level of MCP-1 on day 2 (Figure 7C) [(+) vs M *p* < 0.0001]. The elevated level of MCP-1 was also detected in the serum of MIS416-treated group on day 11. Although MIS416-mediated MCP-1 secretion was decreased in the co-treated group, the level of MCP-1 remained significantly higher than in the mice treated with hUCB-MSCs alone (Figure 7D) [(+) vs M *p* < 0.0001; (+) vs U + M *p* > 0.9999; U vs U + M *p* = 0.0047; M vs U + M *p* < 0.0001]. To address the potential for MCP-1 to stimulate the migratory capacity of hUCB-MSCs, an *in vitro* migration assay was conducted, where MCP-1-primed hUCB-MSCs showed increased migratory capacity (Figure 7E) (U vs U + MCP-1 *p* = 0.0033). These data suggest that MIS416-mediated enhanced MCP-1 secretion may be important for the improved migration of hUCB-MSCs to inflamed colon.

## Discussion

In this study, we have proposed co-administration of MIS416 and MSCs as an enhancement strategy for cell therapy. This strategy may be more convenient for clinical application compared to previous methods (44–46) that require additional preparatory steps, such as cell priming or genetic manipulation. Also, this approach could reduce the associated risks with such additional manipulations, which include tumor formation and contamination of a heterogeneous population. Through gross, histologic, and serologic assessments, we demonstrated that administration of MIS416 distinctly increased the therapeutic effects of hUCB-MSCs in experimental colitis model.

Of note, the improved therapeutic efficiency was not likely mediated by direct interaction between MIS416 and hUCB-MSCs, as we demonstrated that MIS416 treatment could not alter the proliferative, immune cell suppressive, and migratory capacity of hUCB-MSCs. In fact, this finding is not unexpected, due to the physical properties of MIS416, which restrict cell uptake and subsequent sensing of MIS416 ligands to phagocytic innate immune cells, such as plasmacytoid dendritic cells, myeloid dendritic cells, and macrophages (7, 14). Consistent with hUCB-MSCs, CD4^+^ T cells were not directly influenced by MIS416. And MIS416-mediated expansion of immune cells was observed only when innate immune cells were present (7, 14). In the present study, we also demonstrated that MIS416-mediated increased proliferation of hUCB-MNCs was relatively impeded when CD14^+^ macrophage-like cells were depleted.

We showed that application of MIS416 microparticle contributed to increased numbers of immune cells in the spleen. In addition, we examined the effects of MIS416 on the composition of splenic immune cells. Interestingly, proinflammatory cells, including Th1 cells and Th17 cells, were suppressed, and conversely, the proportions of Th2 cells, B cells, and Treg cells were increased. Stimulation of innate immune receptors and their downstream signaling pathways modulated the balance between various immune cells (47, 48). The innate ligands within MIS416 each have well documented immune regulatory activities. NOD2 ligand has been shown to regulate Th1 responses and simultaneously, play a crucial role in the induction of Th2 immune responses and Treg cells (49–51). In addition to this, TLR9 stimulation drives maturation and proliferation of B cells (52, 53). Based on these results, it may be delineated that MIS416 favors the development of regulatory immune cell subsets, although MIS416 induced increases in the quantity of both pro- and anti-inflammatory immune cells. The DSS-induced increase of proinflammatory cytokines, except for IL-6 was attenuated in the serum of MIS416-treated mice on day 11. This suggests that alternative immune cells induced by MIS416 treatment are preferentially able to suppress inflammation and gradually restore immunologic balance in the body.

Imbalance between Th1/Th17 cells and Treg cells in intestine leads to dysregulated inflammation and consequently onset of IBDs (54, 55). MIS416 is known to promote myeloid cell activity and increase the innate IFN-γ to modify disease activity in an auto-immune model (13, 14). In the same context with the previous article (7), the level of IFN-γ was elevated in the serum of MIS416-infused mice. In agreement, Girvan et al. and White et al. have reported that MIS416-mediated innate IFN increased the secretion of TGF-β1 and IL-10, MIS416 also increased the expression of PDL-1 on myeloid cells known to induce the formation of Treg cells (7, 14). Moreover, the type I IFN induced by TLR9 activation suppressed the differentiation of Th1 and Th17 cells, whereas the expansion of Treg cells was promoted (12, 56–58). Consistent with these reports, we observed that treatment with MIS416 decreased the proportion of Th17 cells in hUCB-MNCs and the levels of IL-17A and IL-23 in the serum of mice. In addition, MIS416 increased the colonic infiltration of Treg cells by upregulating IL-10 secretion. Importantly, the levels of IL-17A and IL-23 were mostly reduced in hUCB-MSCs and MIS416 co-treated mice, and the infiltration of Treg cells and IL-10 secretion were notably augmented by co-treatment. Our findings indicate that MIS416 and hUCB-MSCs cooperated to resolve an intestinal inflammation and attenuate the severity of experimental colitis by controlling the balance in Th1, Th17, and Treg cells.

In addition to IFN-γ, the level of IL-6 and IL-12 secretion in the serum of mice was also elevated by MIS416 treatment. Reciprocal interaction between peritoneal immune system and general immune system is accomplished by exchanging the fluids and cells. In addition, peritoneal immune response affects the functions of infused hMSCs, which is attached to specific peritoneal sites, such as mesentery and omentum (39, 40). The combination of these cytokines enhanced the immunosuppressive ability of hUCB-MSCs by upregulating the COX-2- and IDO-1-related pathways (5, 59). Although MIS416 has been reported to increase the level of NO in the serum (14), MIS416-induced cytokines could not alter NO production by hUCB-MSCs. Based on these data, it seems that upregulation of these cytokines plays a role in increased immunomodulation and tissue regeneration by hUCB-MSCs *in vivo*.

Mesenchymal stem cells suppress activated immune cells through direct cell-to-cell contact inhibition as well as environmental change mediated by soluble factors (17, 60). Thus, mobilization of MSCs into inflamed sites is important for suppression of activated immune cells placed in the adjacent lesion. Consistent with previous studies (19, 22, 23), hUCB-MSCs migrated to inflamed colon of mice. More interestingly, this study showed that systemic infusion of MIS416 immediately generated robust production of MCP-1. MCP-1 is known to induce the migration of various types of cells involved in the recovery process, including MSCs (26, 27, 61). MIS416-induced MCP-1 enhanced the migratory capacity of hUCB-MSCs *in vitro* and *in vivo*; thus, many more hUCB-MSCs mobilized to the inflamed colon in response to MCP-1.

Through these findings, we reveal that application of MIS416 ameliorated DSS-induced colitis compared to a single application of hUCB-MSCs and that this effect was mediated through three different ways. First, by inhibition of Th1 and Th17 cells, polarization of Th2 cells, and enhancement of Treg and B cells. In particular, MIS416 and hUCB-MSCs cooperated to shift the balance from Th1/Th17 to the Treg-directed responses. Second, MIS416-mediated changes in immune milieu facilitated the increase of cytokines, such as IFN-γ, IL-6, and IL-12. The hUCB-MSCs stimulated by these cytokines subsequently suppress proinflammatory cells in the inflamed colon. Last, MIS416-induced MCP-1 enhanced the migratory capacity of hUCB-MSCs, resulting in an increase in colonic infiltration. In summary, MIS416 enhances the therapeutic efficacy of hUCB-MSCs against experimental colitis by improving the immunosuppressive capacity of the cells and regulating immune homeostasis in the gut.

## Ethics Statement

All animal experimental processes were approved by Seoul National University Institutional Animal Care and Use Committee (IACUC No. SNU-170523-3) in accordance with the guidelines of the committee. All experiments using human UCB or UCB-derived cells were approved by Institutional Review Board (IRB) of the Boramae Hospital and Seoul National University (IRB No. 1707/001-008) with informed maternal consent. All subjects gave written informed consent in accordance with the Declaration of Helsinki.

## Author Contributions

B-CL and NS designed the study, collected and analyzed the data, and wrote the manuscript. JL, IK, and J-JK collected and analyzed the data. SL collected the data. SC collected and analyzed the data. GW analyzed data and contributed to the writing of the paper. K-SK designed and supervised the study, analyzed the data, and wrote the manuscript.

## Conflict of Interest Statement

The authors declare that the research was conducted in the absence of any commercial or financial relationships that could be construed as a potential conflict of interest.
