# Supplementary material for: MIS416 Enhances Therapeutic Functions of Human Umbilical Cord Blood-Derived Mesenchymal Stem Cells Against Experimental Colitis by Modulating Systemic Immune Milieu
Source: Front Immunol. 2018 May 28;9:1078. doi: 10.3389/fimmu.2018.01078 (PMC5985498; doi:10.3389/fimmu.2018.01078)
Supplement: Supplementary file 1 [file image_1.PDF]

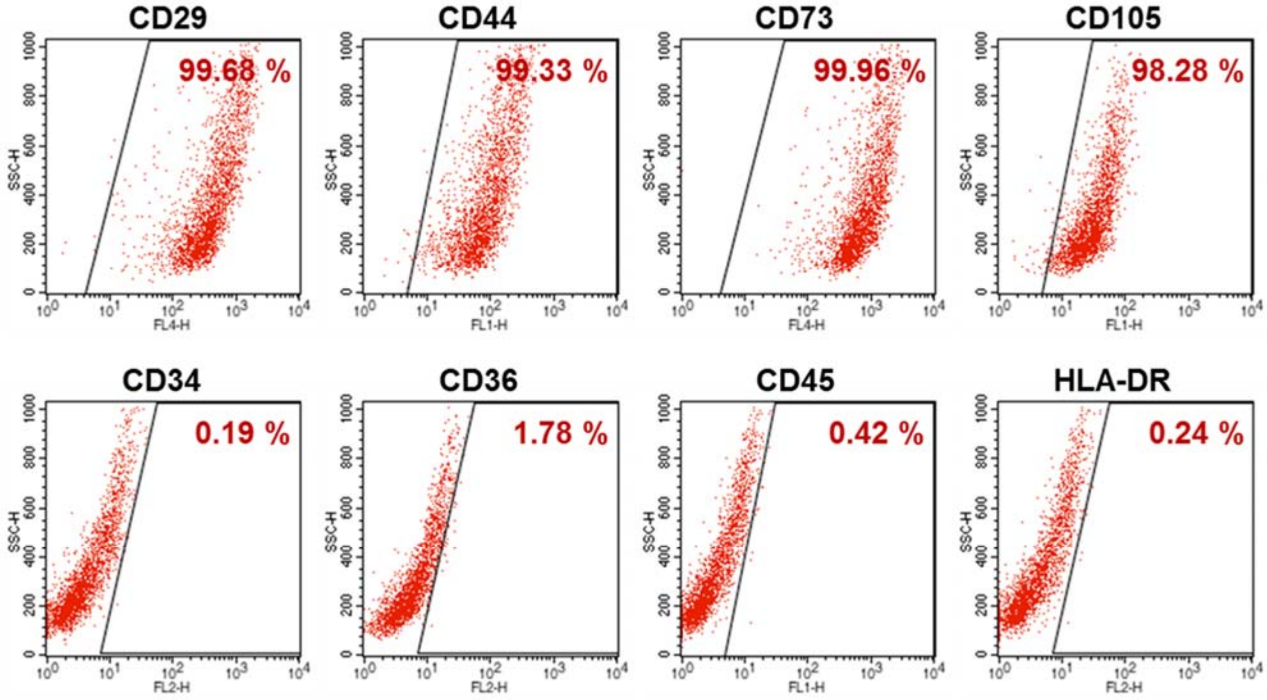

Positive Markers			
CD29	CD44	CD73	CD105
99.59±0.08505	99.29±0.04509	99.94±0.02082	98.05±0.2128
**Negative Markers**			
CD34	CD36	CD45	HLA-DR
0.25±0.07211	2.047±0.6336	0.5067±0.08083	0.2733±0.04163

Supplementary Figure S1. Cell surface marker expression of hUCB-MSCs The expression profile of cell surface markers in hUCB-MSCs were measured by flow cytometric analysis. Positive marker: CD29, CD44, CD73 and CD105, Negative marker: CD34, CD36, CD45 and HLA-DR. Dot plot images show a representative results (upper panel). Results are presented as means ± SEM from three independent experiments (lower panel).
