# Supplementary material for: MIS416 Enhances Therapeutic Functions of Human Umbilical Cord Blood-Derived Mesenchymal Stem Cells Against Experimental Colitis by Modulating Systemic Immune Milieu
Source: Front Immunol. 2018 May 28;9:1078. doi: 10.3389/fimmu.2018.01078 (PMC5985498; doi:10.3389/fimmu.2018.01078)
Supplement: Supplementary file 2 [file image_2.PDF]

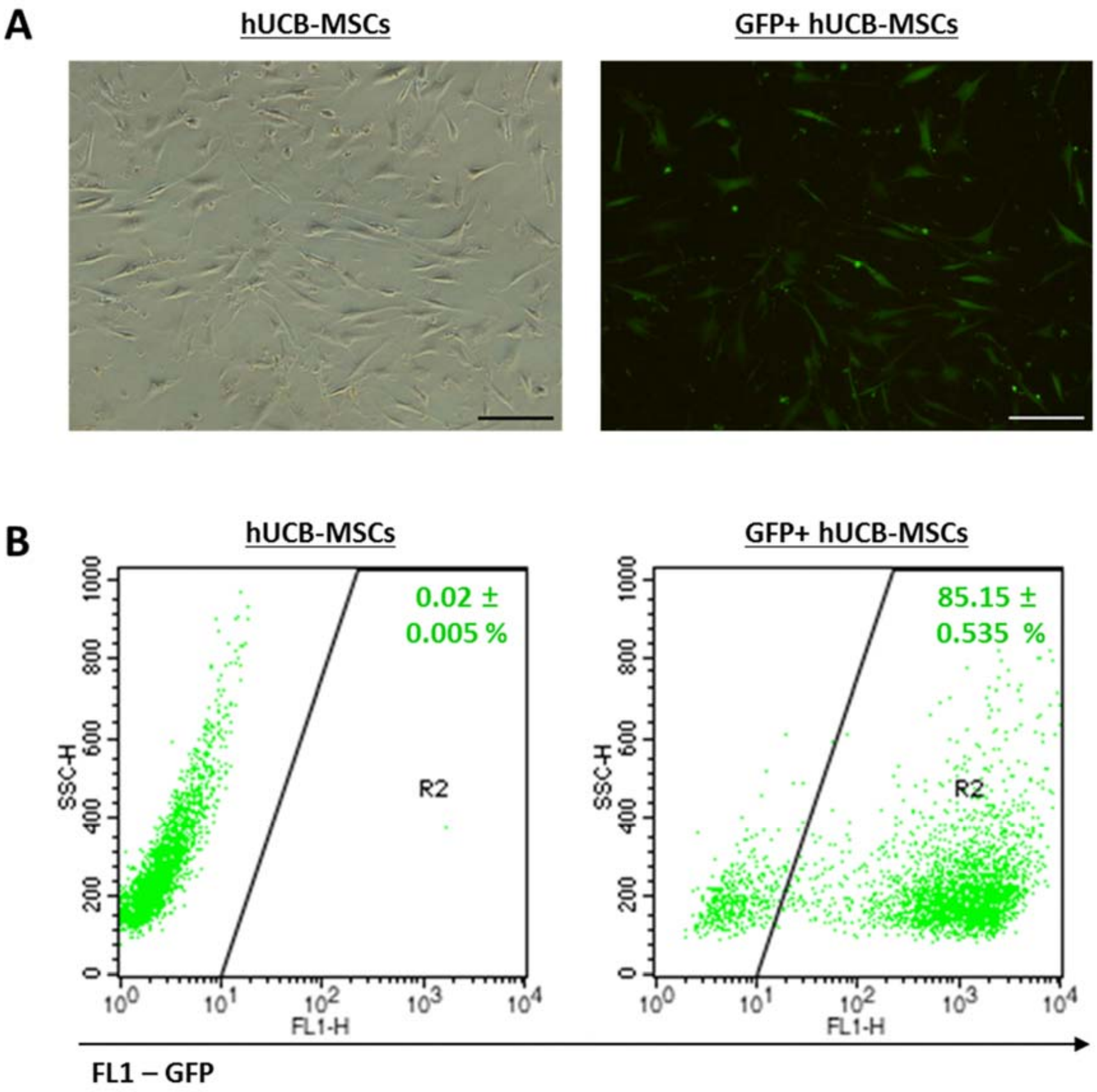


**Supplementary Figure S2. Transduction efficiency of eGFP positive hUCB-MSCs** hUCB-MSCs were transfected using pMX-GFP vector and retrovirus packaging vectors. Transduction efficiencies were determined by (A) bright-field microscopy (left) and fluorescence microscopy (right) of GFP labelled hUCB-MSCs, bar = 100 μm. (B) Representative dot plot images for population of GFP positive cells were determined by flow cytometric analysis. Results are presented as means ± SEM from three independent experiments.
