# Supplementary material for: MIS416 Enhances Therapeutic Functions of Human Umbilical Cord Blood-Derived Mesenchymal Stem Cells Against Experimental Colitis by Modulating Systemic Immune Milieu
Source: Front Immunol. 2018 May 28;9:1078. doi: 10.3389/fimmu.2018.01078 (PMC5985498; doi:10.3389/fimmu.2018.01078)
Supplement: Supplementary file 3 [file image_3.PDF]

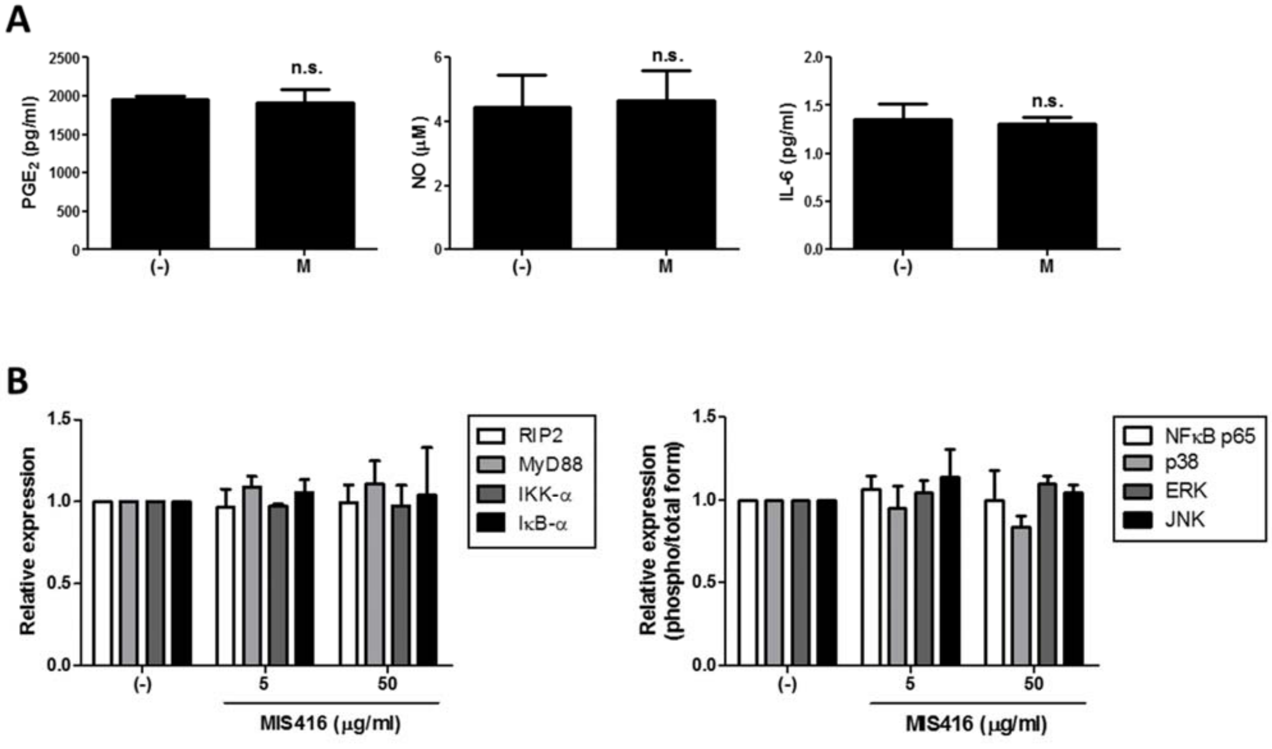

Supplementary Figure S3. Secretory profiles and gene expression patterns of hUCB-MSCs after MIS416 treatment hUCB-MSCs were treated with MIS416 for 24 hours. (A) Secretion levels of PGE_2, NO and IL-6 were measured by ELISA. (50 μg/ml of MIS416) (B) (Left) Expression levels of RIP2, MyD88, IKK-α and IκB-α/ (Right) Phosphorylation levels of NFκB p65, p38 MAPK, ERK and JNK were determined by western blot analysis and quantified. (-): Negative control group, M: MIS416 treated group. Results are presented as means ± SEM from three independent experiments.
