# Supplementary material for: MIS416 Enhances Therapeutic Functions of Human Umbilical Cord Blood-Derived Mesenchymal Stem Cells Against Experimental Colitis by Modulating Systemic Immune Milieu
Source: Front Immunol. 2018 May 28;9:1078. doi: 10.3389/fimmu.2018.01078 (PMC5985498; doi:10.3389/fimmu.2018.01078)
Supplement: Supplementary file 4 [file image_4.PDF]

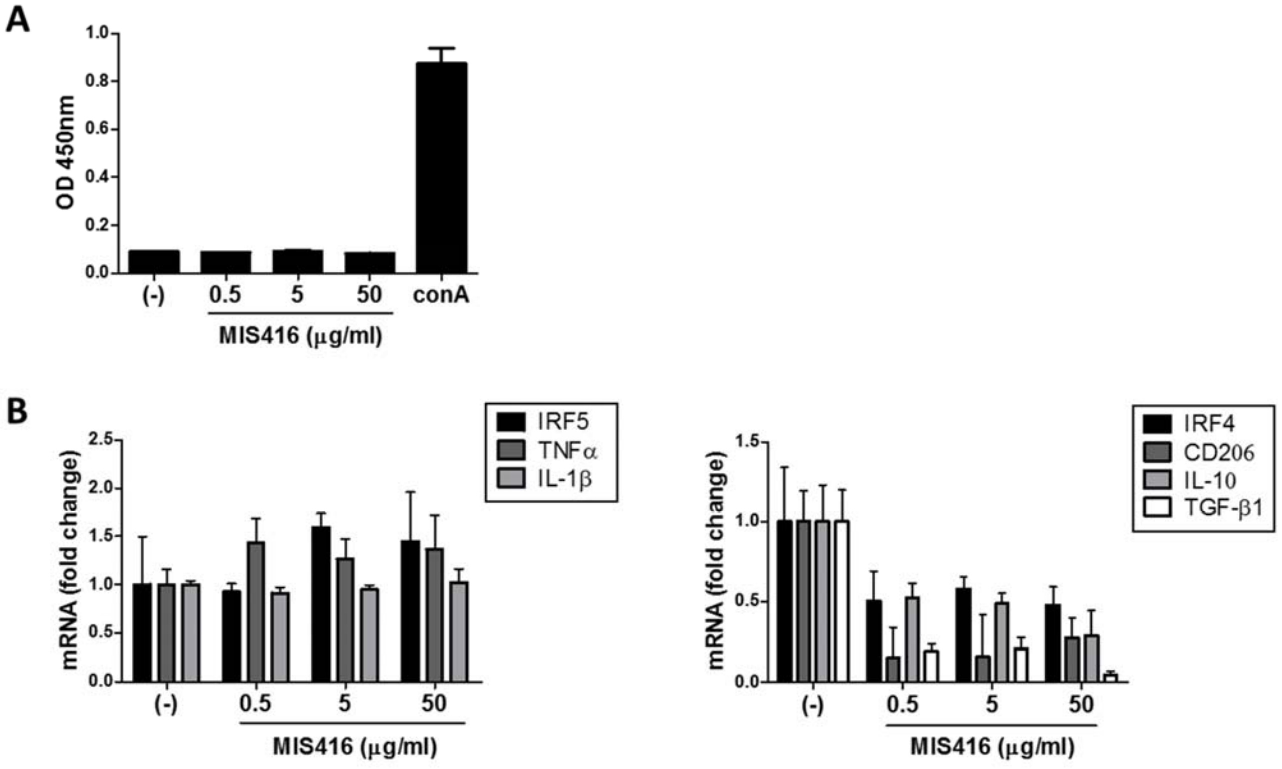


**Supplementary Figure S4. MIS416 influences proliferation and polarization of hUCB-MNCs derived lineage specific immune cells** (A) hUCB-MNCs-derived $CD4^+$ T cells were treated with indicated concentrations of MIS416 for 3 days. Proliferations of isolated $CD4^+$ T cells were measured by BrdU ELISA kit. (B) $CD14^+$ cells were treated with indicated concentrations of MIS416 for 1 day. The mRNA levels of M1 and M2 type markers were analyzed by quantitative PCR. Results are presented as means ± SEM from three independent experiments.
