# Supplementary material for: MIS416 Enhances Therapeutic Functions of Human Umbilical Cord Blood-Derived Mesenchymal Stem Cells Against Experimental Colitis by Modulating Systemic Immune Milieu
Source: Front Immunol. 2018 May 28;9:1078. doi: 10.3389/fimmu.2018.01078 (PMC5985498; doi:10.3389/fimmu.2018.01078)
Supplement: Supplementary file 5 [file image_5.PDF]

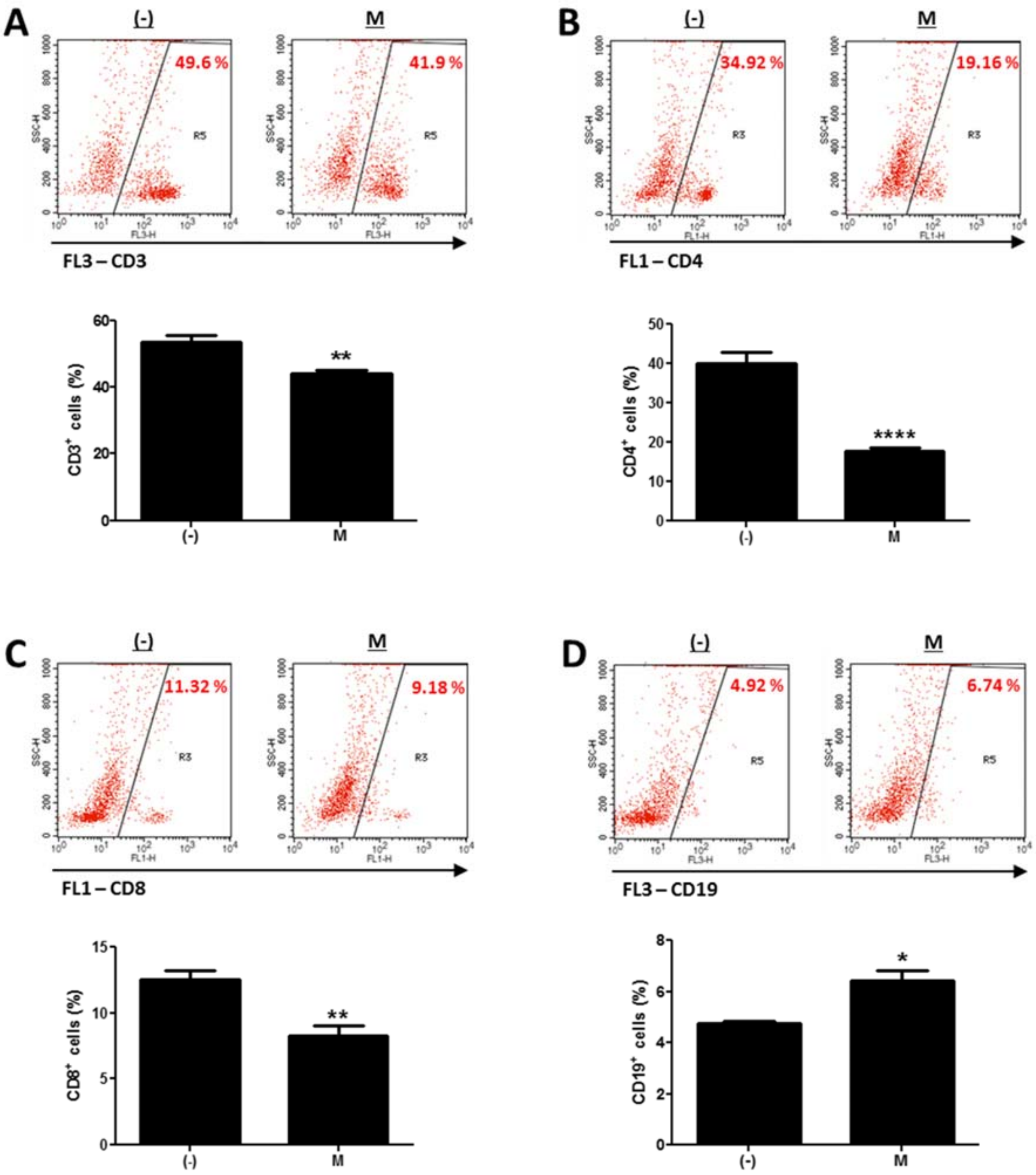

Supplementary Figure S5. MIS416 alters proportion of lineage-specific immune cells hUCB-MNCs were treated with MIS416 (50 μg/ml) for 3 days, and (A) CD3, (B) CD4, (C) CD8 and (D) CD19 expressing cells were analyzed by flow cytometric analysis. (-): Negative control group, M: MIS416 treated group. *P<0.05, **P<0.01, ****P<0.0001. Results are presented as means ± SEM from three independent experiments.
