# Supplementary material for: MIS416 Enhances Therapeutic Functions of Human Umbilical Cord Blood-Derived Mesenchymal Stem Cells Against Experimental Colitis by Modulating Systemic Immune Milieu
Source: Front Immunol. 2018 May 28;9:1078. doi: 10.3389/fimmu.2018.01078 (PMC5985498; doi:10.3389/fimmu.2018.01078)
Supplement: Supplementary file 6 [file image_6.PDF]

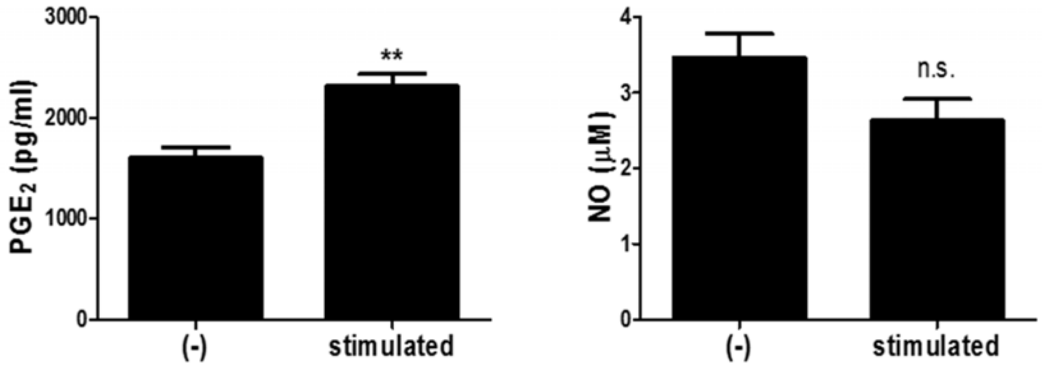

Supplementary Figure S6. Secretory profiles of hUCB-MSCs stimulated by the cytokine cocktail hUCB-MSCs were treated with IL-6 (25 ng/ml), IL-12(20 ng/ml) and IFN-γ (20 ng/ml) for 24 hours. Secretion levels of PGE_2 and NO were measured by ELISA. (-): Negative control group, stimulated: cytokine cocktail-treated hUCB-MSCs. **P<0.01. Results are presented as means ± SEM from three independent experiments.
