# Supplementary material for: MIS416 Enhances Therapeutic Functions of Human Umbilical Cord Blood-Derived Mesenchymal Stem Cells Against Experimental Colitis by Modulating Systemic Immune Milieu
Source: Front Immunol. 2018 May 28;9:1078. doi: 10.3389/fimmu.2018.01078 (PMC5985498; doi:10.3389/fimmu.2018.01078)
Supplement: Supplementary file 7 [file image_7.PDF]

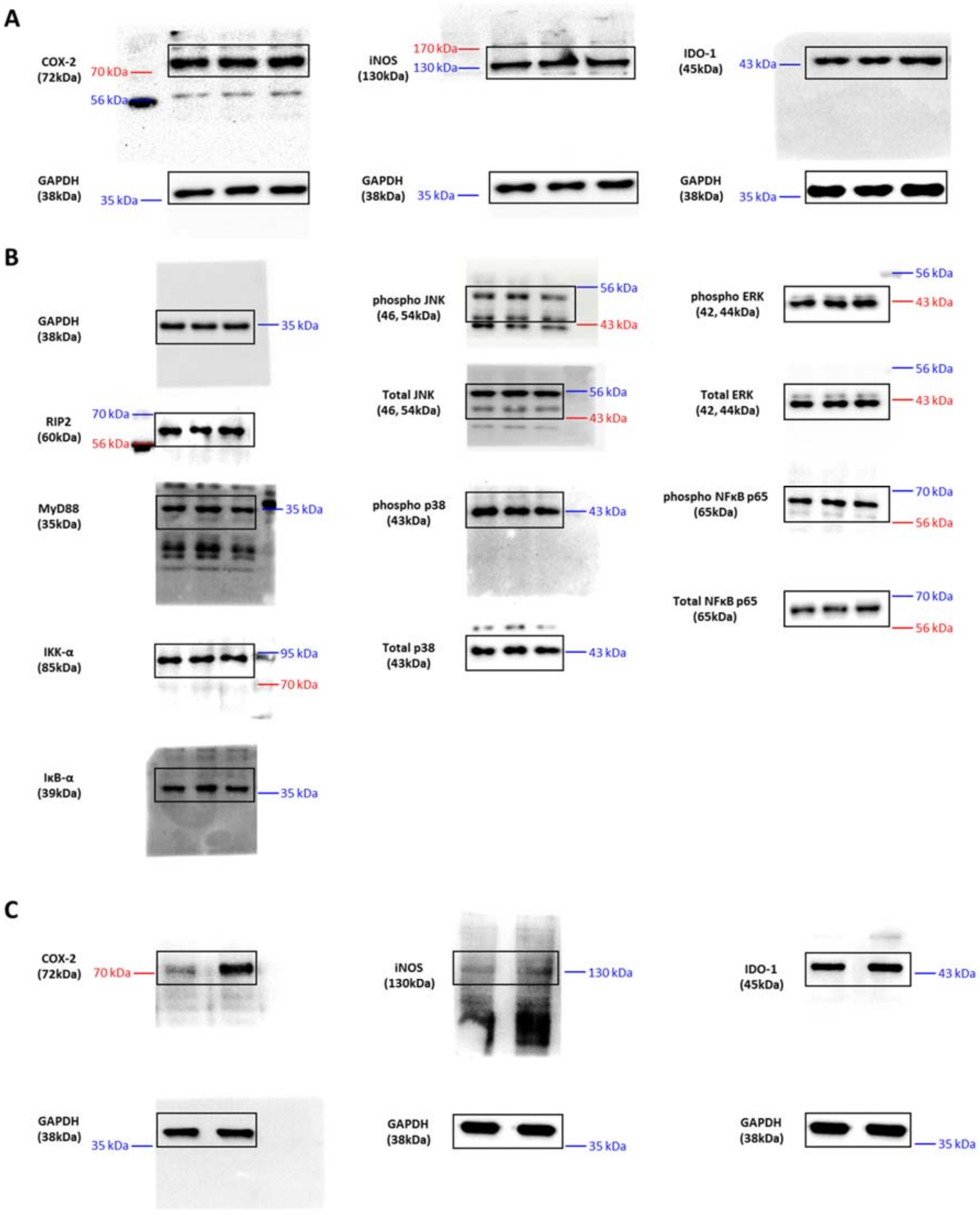

Supplementary Figure S7. Uncropped blot images of key data. Uncropped blot images of (a) Figure 3D, (b) Figure 3E and (c) Figure 6E. Gel electrophoresis was conducted under the same experimental conditions. Cropping lines are indicated.
