# Supplementary material for: MIS416 Enhances Therapeutic Functions of Human Umbilical Cord Blood-Derived Mesenchymal Stem Cells Against Experimental Colitis by Modulating Systemic Immune Milieu
Source: Front Immunol. 2018 May 28;9:1078. doi: 10.3389/fimmu.2018.01078 (PMC5985498; doi:10.3389/fimmu.2018.01078)
Supplement: Supplementary file 8 [file table_1.PDF]

Supplementary Table

	Primary antibody	Dilution	Source	Cat. #
**Western blotting**	Rabbit anti-COX-2	1:1000	Abcam, Cambridge, MA, USA	ab15191
	Mouse anti-iNOS	1:100	Santa Cruz Biotechnology, Santa Cruz, CA, USA	sc-7271
	Mouse anti-IDO-1	1:500	Merck-Millipore, Darmstadt, Germany	MAB5412
	Rabbit anti-RIP2	1:1000	Abcam, Cambridge, MA, USA	ab8428
	Rabbit anti-MyD88	1:1000	Merck-Millipore, Darmstadt, Germany	AB16527
	Rabbit anti-IKK alpha	1:1000	Cell Signaling Technology, Beverly, MA, US	2682s
	Rabbit anti-Phospho NFkB p65	1:1000	Cell Signaling Technology, Beverly, MA, US	3033s
	Rabbit anti-Total NFkB p65	1:1000	Cell Signaling Technology, Beverly, MA, US	8242s
	Rabbit anti-IkB alpha	1:1000	Cell Signaling Technology, Beverly, MA, US	9242s
	Rabbit anti-Phospho JNK	1:1000	Cell Signaling Technology, Beverly, MA, US	9251s
	Rabbit anti-JNK	1:1000	Cell Signaling Technology, Beverly, MA, US	9252s
	Rabbit anti-Phospho p38	1:1000	Cell Signaling Technology, Beverly, MA, US	4511s
	Mouse anti-Total p38	1:500	Merck-Millipore, Darmstadt, Germany	MABS1754
	Rabbit anti-Phospho ERK	1:1000	Cell Signaling Technology, Beverly, MA, US	9101s
	Rabbit anti-Total ERK	1:1000	Cell Signaling Technology, Beverly, MA, US	4695s
**FACS**	FITC Mouse anti-Human CD4	1:50	BD Bioscience, San Jose, CA, USA	555346
	APC Mouse anti-Human IFN-γ	1:50	BD Bioscience, San Jose, CA, USA	554702
	PE Mouse anti-Human IL-4	1:50	BD Bioscience, San Jose, CA, USA	554516
	PE Mouse anti-Human FoxP3	1:50	BD Bioscience, San Jose, CA, USA	560046
	PE Mouse anti-Human IL-17A	1:50	BD Bioscience, San Jose, CA, USA	560438
	APC Rat anti-mouse CD4	0.125 µg/test	eBioscience, San Diego, CA, USA	17-0042-82
	PE Rat anti-mouse CD25	0.125 µg/test	eBioscience, San Diego, CA, USA	12-0251-82
	FITC Rat anti-mouse FoxP3	1 µg/test	eBioscience, San Diego, CA, USA	11-5773-82
	FITC Mouse Anti-Human CD45	1:50	BD Bioscience, San Jose, CA, USA	555482
	PE Mouse Anti-Human HLA-DR	1:50	BD Bioscience, San Jose, CA, USA	555812
	FITC Mouse anti-Human CD105 (Endoglin)	1:50	BD Bioscience, San Jose, CA, USA	561443
	APC Mouse anti-Human CD73	1:50	BD Bioscience, San Jose, CA, USA	560847
	APC Mouse Anti-Human CD29	1:50	BD Bioscience, San Jose, CA, USA	559883
	FITC Mouse Anti-Human CD44	1:50	BD Bioscience, San Jose, CA, USA	555478
	PE Mouse Anti-Human CD36	1:50	BD Bioscience, San Jose, CA, USA	555455
	PE Mouse Anti-Human CD34	1:50	BD Bioscience, San Jose, CA, USA	555822
**IHC**	FOXP3 Monoclonal Antibody	1:200	eBioscience, San Diego, CA, USA	14-5773-80

Supplementary Table S1. Antibody information All antibodies used for western blotting, flow cytometry and immunohistochemistry were specified with dilution ratios, sources and catalog numbers.
